# Coupling Enhancement of a Flexible BiFeO_3_ Film-Based Nanogenerator for Simultaneously Scavenging Light and Vibration Energies

**DOI:** 10.1007/s40820-022-00943-0

**Published:** 2022-10-06

**Authors:** Xiao Han, Yun Ji, Li Wu, Yanlong Xia, Chris R. Bowen, Ya Yang

**Affiliations:** 1 CAS Center for Excellence in Nanoscience, Beijing Key Laboratory of Micro-Nano Energy and Sensor, Beijing Institute of Nanoenergy and Nanosystems, Chinese Academy of Sciences, Beijing, 101400 People’s Republic of China; 2 Department of Mechanical Engineering, University of Bath, Bath, BA27AK UK; 3 School of Nanoscience and Technology, University of Chinese Academy of Sciences, Beijing, 100049 People’s Republic of China; 4 Center On Nanoenergy Research, School of Physical Science and Technology, Guangxi University, Nanning, 530004 People’s Republic of China

**Keywords:** Ferroelectric film, Coupled nanogenerators, Photovoltaic effect, Flexoelectric effect, Energy collection

## Abstract

Coupled nanogenerators have been a research hotspot due to their ability to harvest a variety of forms of energy such as light, mechanical and thermal energy and achieve a stable direct current output. Ferroelectric films are frequently investigated for photovoltaic applications due to their unique photovoltaic properties and bandgap-independent photovoltage, while the flexoelectric effect is an electromechanical property commonly found in solid dielectrics. Here, we effectively construct a new form of coupled nanogenerator based on a flexible BiFeO_3_ ferroelectric film that combines both flexoelectric and photovoltaic effects to successfully harvest both light and vibration energies. This device converts an alternating current into a direct current and achieves a 6.2% charge enhancement and a 19.3% energy enhancement to achieve a multi-dimensional "1 + 1 > 2" coupling enhancement in terms of current, charge and energy. This work proposes a new approach to the coupling of multiple energy harvesting mechanisms in ferroelectric nanogenerators and provides a new strategy to enhance the transduction efficiency of flexible functional devices.

**Supplementary Information:**

The online version contains supplementary material available at 10.1007/s40820-022-00943-0.

## Highlights

- Development of new form of coupled nanogenerators to enhance performance.
- Energy in the form of light and vibration is successfully harvested, by converting an alternating current signal into a direct current signal.
- New approach to creating coupled nanogenerators, where multiple harvesting mechanisms by exploiting ferroelectric materials are achieved.

**Supplementary Information:**

The online version contains supplementary material available at 10.1007/s40820-022-00943-0.

## Graphical abstract

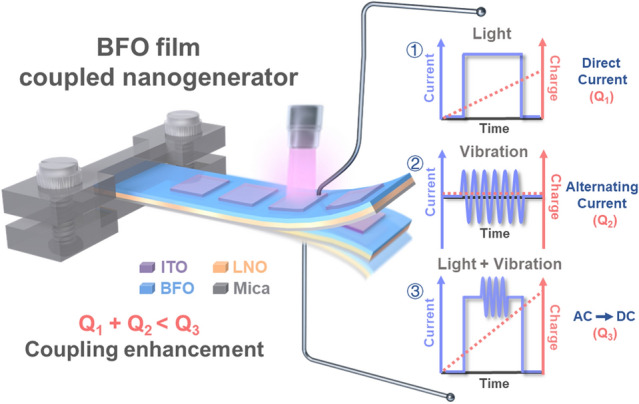

## Introduction

The demand for functional devices for collecting sustainable energy is constantly growing and, as a result, self-powered devices that enable the harvesting of different forms of energy from the ambient environment are of significant technological and industrial relevance [1–6]. Ferroelectric materials are able to generate a voltage without Shockley–Queisser limits to provide distinctive optoelectronic properties and conversion mechanisms [7–9]. Furthermore, the design of transparent ferroelectrics broadens the range of applications of ferroelectric materials to produce self-powered photovoltaic functional devices [10]. Among them, BiFeO_3_ (BFO) with its high remnant polarization and small band gap has gained significant attention in the field of ferroelectric photovoltaics [11–14]. In addition, the flexoelectric effect refers to a polarization induced by a strain gradient within the material, which generates an electrical signal [15]. Flexoelectric effects, which are typically ignored at the macroscopic scale, play a significant role at the nanoscale, where the polarization and domain structure within ferroelectric films can be modulated, and the local photoelectric properties of the material may be altered [16–19]. In addition, thin films can exhibit flexoelectric effects at the macroscopic scale [20], providing the groundwork for the combination of ferroelectric and flexoelectric effects, but limited studies have been conducted on energy harvesting utilizing flexoelectric effects [21].

Photoelectric and electromechanical conversion are two conventional approaches to harvesting energy; however, most materials are only able to harvest a single form of energy which restricts the further development of systems for energy scavenging, self-powered sensing and storage. As a multifunctional material, ferroelectric materials are able to generate various types of signals as a result of photoelectric, flexoelectric, thermoelectric and pyroelectric effects [22–26]. Coupled nanogenerators based on inorganic ferroelectric ceramics, including BaTiO_3_, are able to couple photovoltaic and pyroelectric effects [22, 27]. The output electrical signals as a result of synergistic coupling effects are significantly enhanced compared to those of the photovoltaic effect alone, and there is potential to integrate various forms of energy to solve the problems of harvesting a single form of energy; this includes low efficiency, the intermittency of a single energy supply, and high cost [28, 29]. Due to the excellent photovoltaic performance of lead-free BFO ferroelectric films, coupling ferroelectric photovoltaics with other external input such as temperature, strain and an electric field is of particular interest in the industries of nano-energy and self-powered sensing [22, 30]. In addition, the introduction of oxygen vacancies provides greater possibilities for tailoring ferroelectric photovoltaic effects in BFO films, offering new ideas for the advanced design of switchable devices [31]. Based on this knowledge, in this paper we exploit the photovoltaic and flexoelectric signals generated by a BFO ferroelectric film, and by careful control of the interaction between the two signals we are able harvest both light and vibration energies, where the energy harvesting efficiency and signal responsiveness of the device are effectively enhanced.

Unlike the strain gradients associated with material deposition or scanning probe microscopy [32], we achieve continuous regulation of the strain gradient in BFO films by deposition on a mica substrate and altering the bending radius of the flexible film. We have engineered the 4 × 1 rectangular array based on BFO ferroelectric film to effectively collect vibrational and light energy, and to achieve the "1 + 1 > 2" charge enhancement at different wavelengths, where the charge collected by the photoelectric-flexoelectric coupling is more than that of the two mechanisms operating individually. At the same time, the photoconductive gain and responsiveness of the device are increased by 25–50% and we demonstrate the successful harvesting of vibrational energy that is manifested by alternating current (AC) signals, which is of significance for the simultaneous harvesting of light and vibration energies from the environment. This new approach demonstrates the significant potential of ferroelectric materials for coupled nanogenerators.

## Experimental Section

### Materials and Reagents

Bi(NO_3_)_3_·5H_2_O and La(NO_3_)_3_·6H_2_O were provided by Shanghai Aladdin Biochemical Technology Co., Ltd., and Fe(NO_3_)_3_·5H_2_O, Ni(CH_3_COO)_2_·4H_2_O, 2-methoxyethanol, acetic acid, ethanolamine, and ethanol anhydrous were purchased from Shanghai Macklin Biochemical Co., Ltd. Acetic anhydride was purchased from Sinopharm Chemical Reagent Co., Ltd.

### Precursor Fluid Preparation

The BiFeO_3_ ferroelectric films are prepared by a simple sol–gel method. A mass of 1.6169 g of Bi(NO_3_)_3_·5H_2_O, and 1.212 g of Fe(NO_3_)_3_·5H_2_O were dissolved in a mixture of 2-methoxyethanol and acetic acid, and then mixed. Acetic anhydride was added as the dehydrating agent, finally, 100 μL of ethanolamine was added to adjust the viscosity of the solution, and finally, 10 mL of precursor solution (0.3 mol L^−1^) was obtained by adding solvent, stirring for 24 h.

LaNiO_3_ films were prepared as the bottom electrode via a similar method. A mass of 1.4945 g of Ni(CH_3_COO)_2_·4H_2_O was dissolved in 12 mL of acetic acid, and 2.5983 g of La(NO_3_)_3_·6H_2_O was added to the dissolved solution and finally, 30 mL of ethanol anhydrous was added and stirred for 10 min to obtain the LaNiO_3_ solution.

### Construction of LNO/BFO/ITO

The aged LNO solution and the BFO precursor solution were spin-coated sequentially onto a clean mica substrate by a spin coater. The LNO was spin-coated at 2000 rpm for 20 s and heated sequentially on a hot plate at 180, 400 and 700 °C for 3 min. The above process was repeated six times. The BFO was spin-coated at 4000 rpm for 15 s and heated on a hot plate at 200, 400 and 550 °C for 3, 3 and 10 min, respectively, and the process was repeated two times to obtain BFO films. The indium tin oxide (ITO) top electrode was deposited onto the mica/LNO/BFO film using magnetron sputtering equipment (MSP-820) and a 2 × 2 mm mask plate.

### Performance Measurement and Characterization

A light-emitting diode (LED) light provided the incident light and a power meter (OPHIR) was used to detect the light power density. A vibration exciter (Labworks inc.) applied vibration to the device. A Keithley 2611B based system source meter was used to measure output signals.

A scanning electron microscope (SU8020 cold field SEM) and an AFM atomic force microscope (MFP-3D-SA) were used to observe the morphology of the BFO films. XRD (Philips, Panalytical X'pert3, Cu Kα radiation source) was used to characterize the phase and crystal structure. A ferroelectric materials test system (RTI-MultiFerroic) was used for the ferroelectric hysteresis loop testing. A piezoelectric force microscopy (MFP-3D-SA) was used for ferroelectric domain characterization. A confocal micro-Raman spectrum (LABRAM HR EVOLUTION) was used for Raman spectrum characterization of ferroelectric films. A UV–Vis–NIR spectrophotometer (UV3600) was used for the absorption spectrum testing.

## Results and Discussion

### Structure and Material Characterization of Devices

**Fig. 1:**
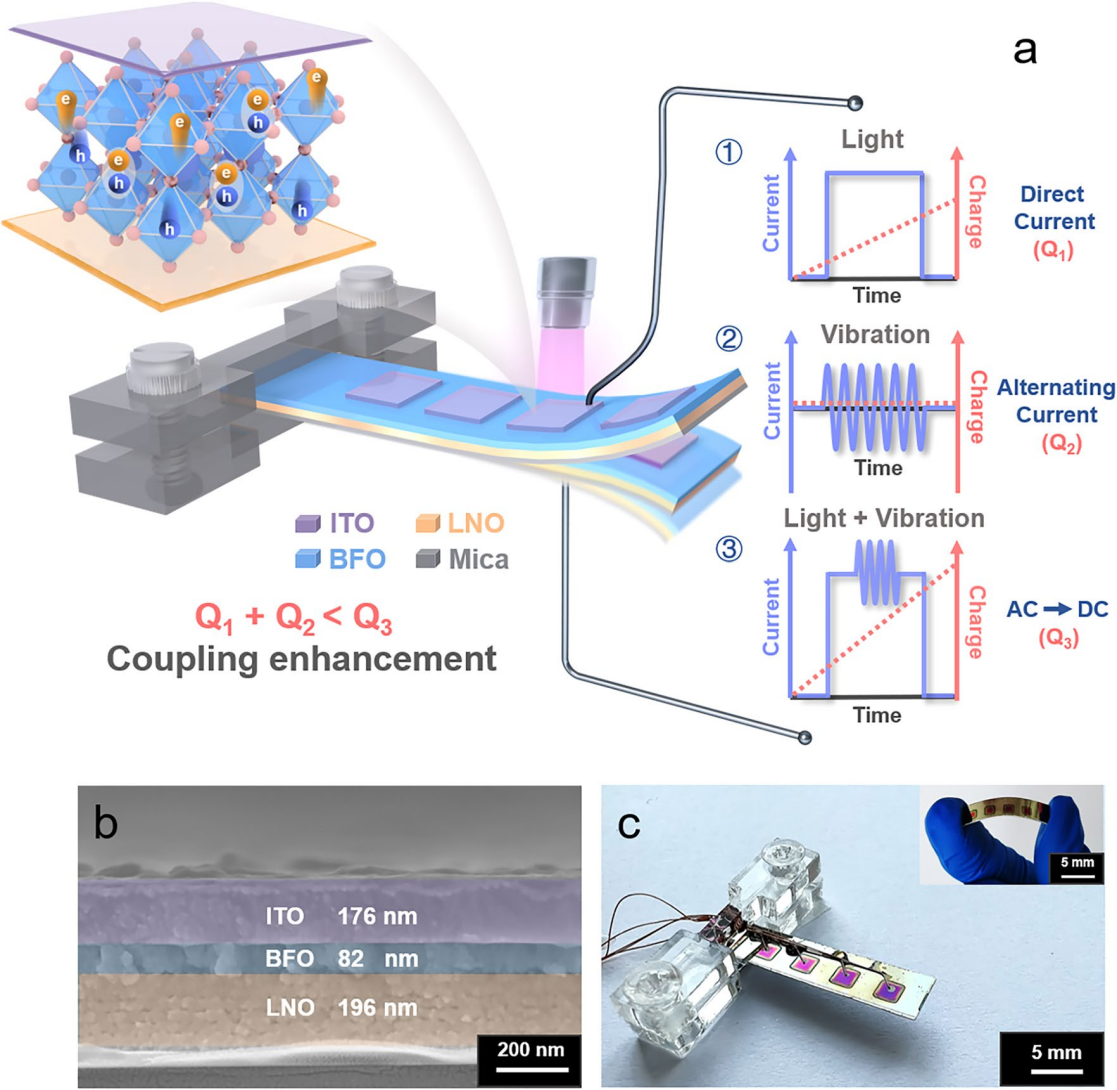
Design of LNO/BFO/ITO nanogenerator for scavenging energies. **a** Schematic of the operation of the coupled nanogenerator based on LNO/BFO/ITO and the corresponding signal schematic. **b** SEM image of the cross section of the device. **c** Optical image of the device, inset schematic showing the device after bending

To efficiently harvest both light and vibration energy, we utilized a vibration exciter to deliver vibrational forces to the free end of a clamped cantilever-shaped film with a LNO (bottom electrode)/BFO (ferroelectric film)/ITO (top electrode) structure. The specially designed structure results in mechanical vibration inducing inhomogeneous deformations and strain gradients into the film. Optical photographs of the device vibrating and bending with the vibration exciter are shown in Fig. S27. The device undergoes significant bending during vibration, and the inhomogeneous deformation is the direct cause of the induced flexoelectricity [30, 33]. The operating state when light and vibration was applied individually or simultaneously to the BFO ferroelectric film is shown in Fig. 1a; the corresponding current signals and charge integration data are also illustrated schematically. At different operating conditions, the carriers within the ferroelectric are effectively separated and transported under the effect of the internal electric field, and the corresponding electrical signals are measured externally. When vibration acts alone, the nanogenerator is unable to collect charge productively since the dynamic loads due to the vibrations generates an AC around the "0" baseline and the statistically integrated charge is zero [20]. When the device is exposed to light, incident photons are converted to removable carriers to allow an electrical signal to be generated and measured effectively externally, where the current signal integration corresponds to the level of photogenerated charge. When vibration and light are applied to the device simultaneously, the coupling current is determined by the combined effect of the additional built-in electric field resulting from the flexoelectric effect and the polarization-related internal electric field due to the interaction of photoelectric and flexoelectric effects. Here, more electrons and holes are transferred, resulting in a stronger coupling of current and corresponding increase in statistically integrated charges, achieving a coupling enhancement of "1 + 1 > 2" and the conversation of AC signals to DC signals.

The coupled nanogenerators consist of LNO/BFO/ITO array units grown on a flexible and high-temperature resistant mica substrate. Each array cell was made of LNO ~ 200 nm thickness, BFO ~ 80 nm and an ITO pattern array ~ 180 nm thickness (Fig. 1b). The continuously variable bending radius allows for controlled flexoelectricity, while a treated polyester (PET) film was bonded to the bottom of the device as a protective layer and to provide flexibility and stability, and a weight of 0.4126 g was attached to the end of the device to achieve an effective vibration amplitude and energy scavenging (Fig. 1c).

To observe the morphology of the sample surface, the annealed film surface was observed by scanning electron microscopy (SEM) and atomic force microscopy (AFM). Dense and relatively uniformly sized crystalline grains were observed with a flat surface with almost no defects, with a high-quality film providing the basis for vibration harvesting (Fig. S1a, g, h). Meanwhile, the diffraction peaks corresponding to the X-ray diffraction (XRD) images in the BFO films indicate a rhomboidal phase of the BFO nanoparticles, with atoms deviating from the body center structure and the material showing spontaneous polarization (Fig. S1c). To further confirm the structure of the material, the Raman spectrum of the BFO film was measured and the results of Fig. S1b were almost similar to those of Kothari et al. for the Raman spectrum of the BFO with a R3c structure [34]. The results of the polarization – electric field (P-E) loop demonstrate the ferroelectricity and the direction of spontaneous polarization within the material (Fig. S1e). We have tested the ferroelectric domain structure of the BFO film (Fig. S1f) by piezoelectric force microscopy (PFM), and the reversal of its PFM phase diagram is further evidence of ferroelectricity in the BFO film.

### Photoelectric and Flexoelectric Properties

**Fig. 2:**
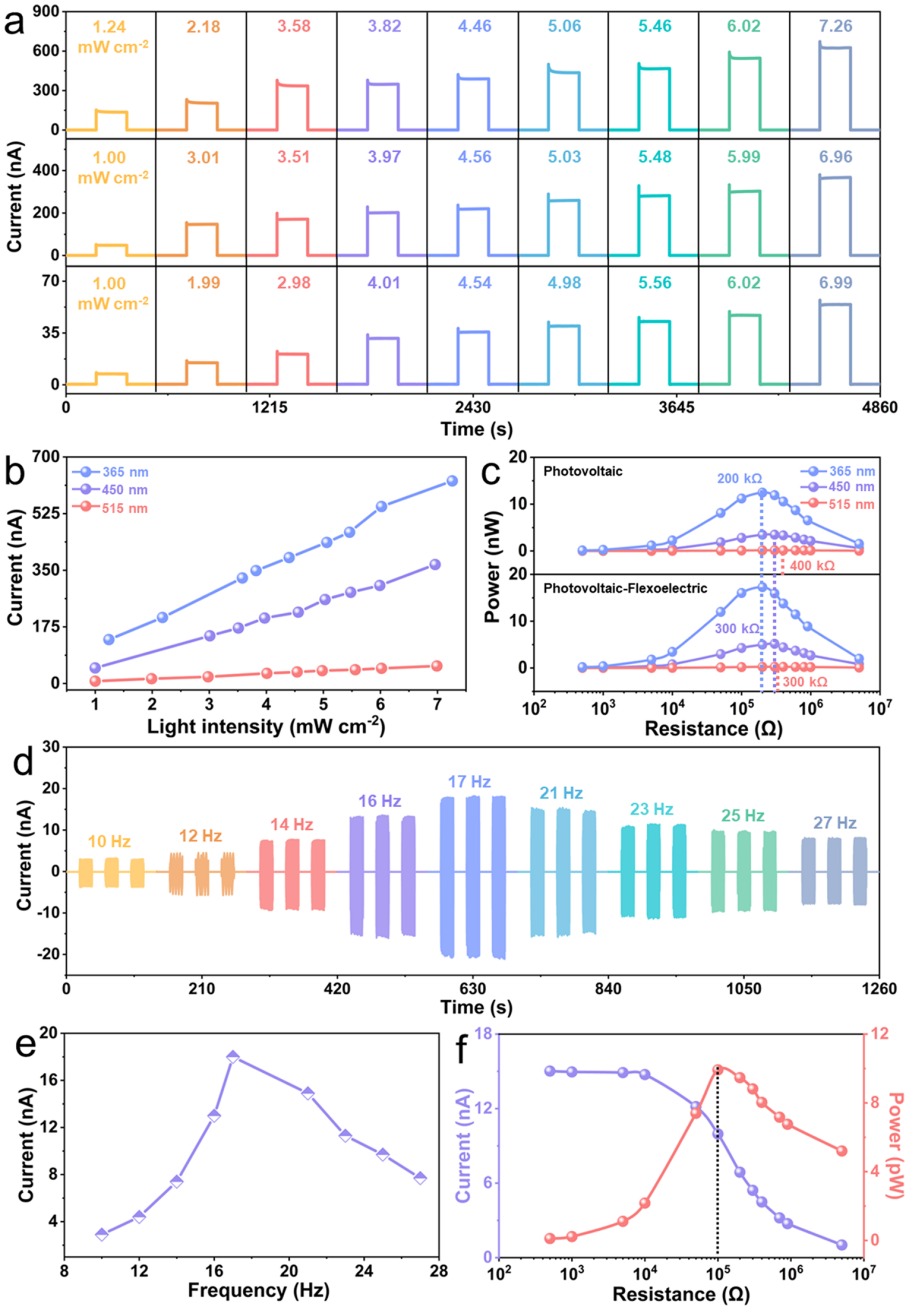
Photovoltaic and flexoelectric performance of the LNO/BFO/ITO device. **a** Short-circuit currents for devices illuminated with different light intensities at 365, 450 and 515 nm. **b** Output current of the device as a function of the light intensity caused by the photoelectric effect. **c** Load-dependent photovoltaic, photovoltaic-flexoelectric output power for devices (4.5 mW cm^−2^). **d** Output current of the device at different vibration frequencies. **e** Output current of a device as a function of frequency caused by the flexoelectric effect. **f** Load-dependent output current and power

An eco-friendly and lead-free multiferroic BFO film was used for this work since it has attractive ferroelectric and photovoltaic properties. With a direct band gap of approximately 2.62 eV, the BFO films demonstrate excellent sensitivity in the UV–Vis range with a broad absorption in the 300–500 nm range, absorbing large amounts of UV–Vis photons (Fig. S1b) and offering a unique opportunity for photovoltaic applications. The photovoltaic and flexoelectric performance of the ITO/BFO/LNO coupled nanogenerator under different operating conditions is shown in Fig. 2. The output signals of the device exposed to different wavelengths of light are illustrated in Fig. S2a, b. Three different wavelengths of light, namely 365, 450 and 515 nm, were selected for representative testing (Figs. 2a and S2c–e). The current response with time shows a linear increase when the light is switched on/off at different light intensities, and the current response diagram, depending on the light intensity, demonstrates a strongly linear response (Fig. 2b); this indicates that the ferroelectric photovoltage of BFO ferroelectric films is reproducible, providing strong potential for applications in photodetection. A range of load resistances were used to ascertain the maximum output power (Fig. S14) of the ITO/BFO/LNO device by matching the impedance of the device and electrical load, and Fig. 2c shows the corresponding output power (4.5 mW cm^−2^) calculated according to *P* = *I*^2^*R*. As the excitation energy of the photons change, the devices achieve its maximum output power of the photoelectric and photoelectric-flexoelectric coupling with different resistances at different wavelengths. The results reveal that the maximum output power of the device caused by the photoelectric-flexoelectric coupling is significantly higher than the sum of that caused by the photoelectric effect and the ferroelectric alone (Fig. 2f); therefore, the interaction of the two effects effectively enhances the output power of the device.

The application of mechanical vibrations and resulting bending introduces strain gradients and generates an AC current due to the dynamic forces. A movie of the device in operation with bending occurring is shown in Movie S1. The BFO ferroelectric films exhibit a frequency and amplitude dependence under the influence of external forces, where different bending radii correspond to different values of flexoelectric signals (Figs. 2d and S3). As the frequency varies from 10 to 27 Hz, the corresponding flexoelectric output current and voltage initially increases and then decreases. At the maximum amplitude and frequency of 17 Hz, the BFO film achieves maximum bending deformation and a maximum flexoelectric signal with a current of 18 nA and a voltage of 3.25 mV (Figs. 2e and S3a), and periodic cycling testing of the flexoelectric signal shows excellent repeatability. Similarly, a range of load resistances are used to obtain the maximum instantaneous output power for the flexoelectric signals, with the device achieving maximum output power at a load resistance of 100 kΩ (Fig. 2f). When the difference in values between the photoelectric signal and the flexoelectric signal is small, the resistance used to define the maximum output power is equally important for both signals. Thus, when the device is illuminated at 515 nm, the output power of the coupling signal of the device corresponds to a different optimum load resistance compared to that of the photovoltaic signal alone.

### Coupling Performance of the Nanogenerator

**Fig. 3:**
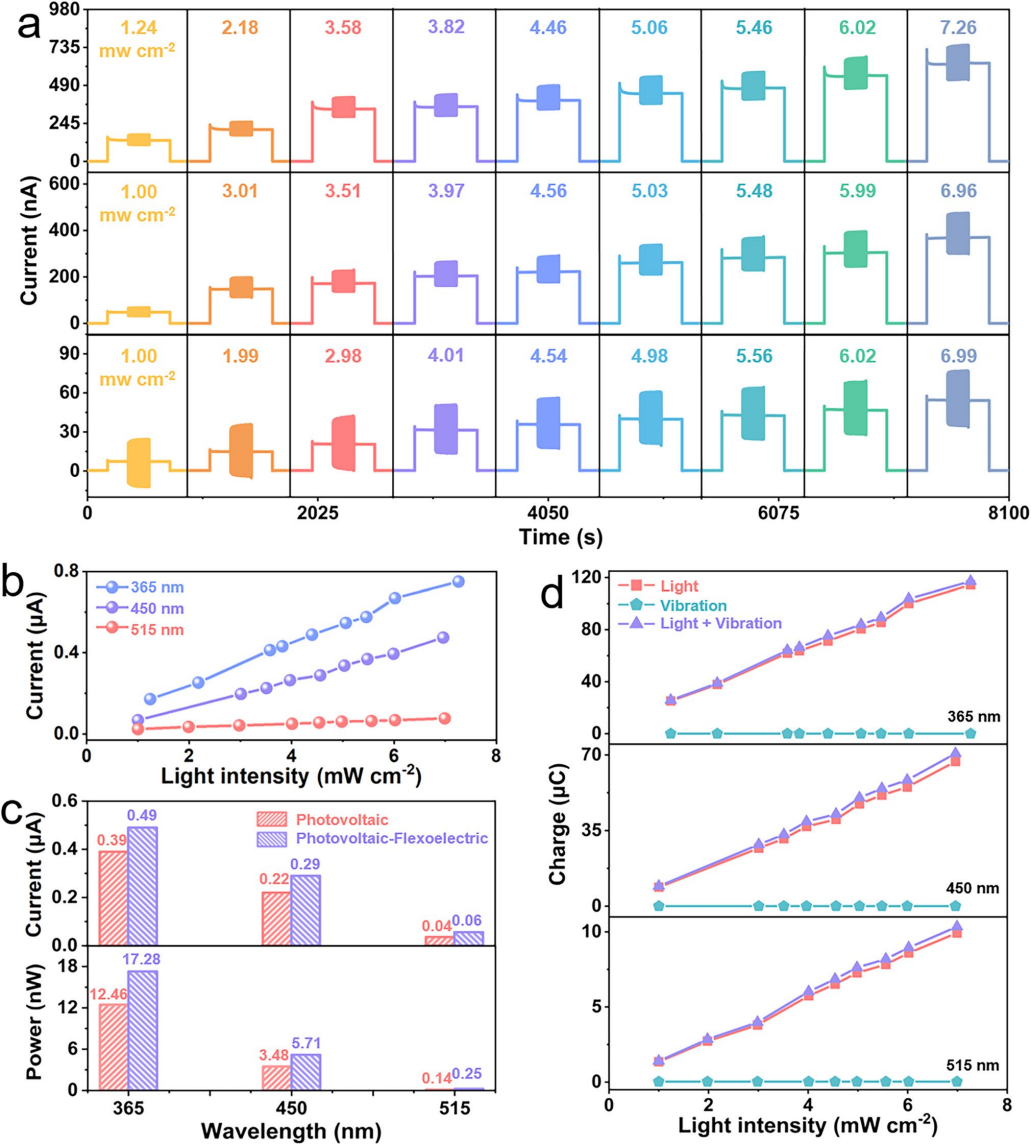
Influence of photoelectric-flexoelectric coupling effects on the electrical properties of LNO/BFO/ITO. **a** Short-circuit current variation in devices caused by light-vibration coupling. **b** Output current of the device as a function of light intensity caused by the photoelectric-flexoelectric effect. **c** Comparison of short-circuit currents and output powers of devices caused by light and light-vibration coupling (4.5 mW cm^−2^). **d** Comparison of collected charges under different working conditions

We further investigated the interaction of photoelectric and flexoelectric effect, where the experimental test scheme is shown in Fig. 1a and the electrical signals of the device when subjected to light, vibration or combined light/vibration are shown in Fig. 3. The variation of the coupling currents of the devices at the three wavelengths of light is shown in Fig. 3a. Both photovoltaic and coupling currents grow sequentially with increasing light intensity, and the coupling effect has a significantly larger current than the photovoltaic effect under the same conditions. Similar, the linearity of the coupling current with increasing light intensity provides an indication of its sensitivity (Fig. 3b), indicating that the flexoelectric effect develops an effective additional electric field that modulates the photocurrent/photovoltage of the BFO ferroelectric film.

The ratio of the number of charges gathered by the electrode to that of photons absorbed by the film in a unit of time is defined as the *photoconductive gain*, defined a *G* = (*I*/*e*)/(*PS*/*hv*), where *I*, *e*, *P*, *S* and hv are the output current, charge, light intensity, effective area and photon energy of the incident light, respectively [35]. Due to the enhanced photoelectric effect as a result of the flexoelectric effect, a 25–50% increase in photoconductive gain is achieved in the coupled state in comparison with that of light alone. Based on a light intensity of 4.5 mW cm^−2^, a comparison of current and output power due to the photovoltaic effect alone and the coupling of photovoltaic-flexoelectric effects at different wavelengths is shown in Fig. 3c. The significant increase in signal under combined light and vibration further demonstrates the effective modulation and enhancement of the photovoltaic effect through the flexoelectric effect. Furthermore, in the course of our study, we found that the interaction of photovoltaic and flexoelectricity leads to a "1 + 1 > 2" charge enhancement when the ferroelectric film is exposed to both light and vibration. By integrating the output currents for independent test times (180 s, Fig. 3d), we can observe that the statistically integrated charges of the device are significantly larger under a combined photoelectric-flexoelectric condition compared to when subjected to light or vibration alone, and the experimental results for different wavelengths confirm the deterministic and reproducible nature of the coupling enhancement. The specific current signals and collected charges for each condition are shown in the supporting file (Figs. S11–S13).

To evaluate the coupling performance of nanogenerators, here we define the ratio of charge enhancement of the device *E (%)* and the Yang coupling factor *K*_*C*_, respectively, where *E (%)* is defined as:

1 $$E\left( \% \right) = \left[ {Q_{{{\text{photo }} - {\text{ flexo}}}} - Q_{{{\text{photo}}}} - Q_{{{\text{flexo}}}} } \right]/\left( {Q_{{{\text{photo}}}} + \, Q_{{{\text{flexo}}}} } \right)*100\%$$

**Fig. 4:**
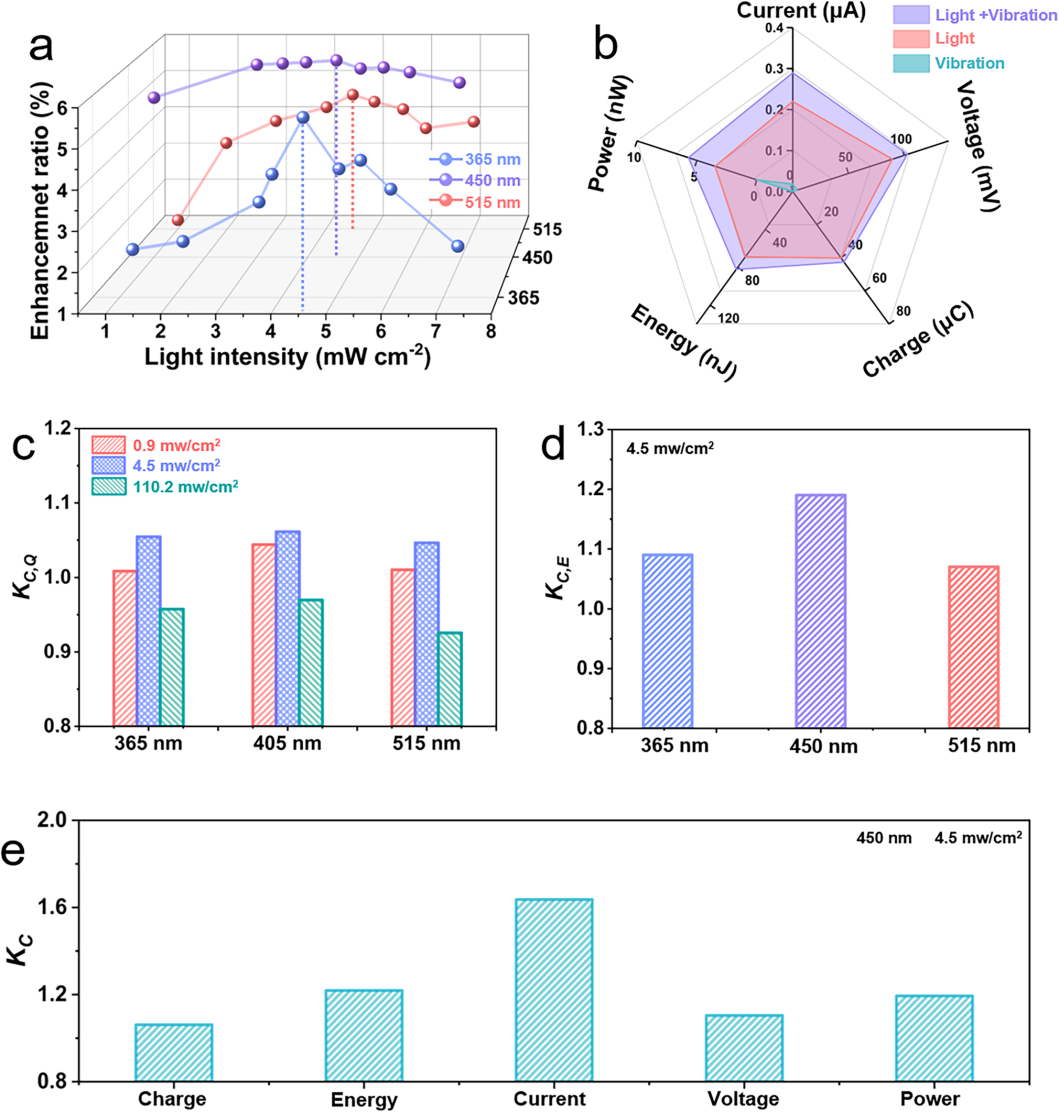
Performance evaluation of LNO/BFO/ITO photoelectric-flexoelectric coupling. **a** Charge enhancement ratio E as a function of light intensity. Comparison of **b** signal and **c**–**e** Yang coupling factor of the device under 450 nm illumination (4.5 mW cm^−2^). Yang coupling factors of **c ***K*_**C, Q**_ charges and **d** *K*_**C, E**_ energies

At a small light intensity, the statistically integrated charges due to the coupling effect are significantly larger than the algebraic sum of the two, *E* > 0. Interestingly, we discovered that the approximate range of light intensities which correspond to the peak charge enhancement is not affected by the wavelength, with the maximum value of *E* falling within the light intensity range of 4–5 mW cm^−2^ at all three wavelengths (Fig. 4a). When exposed to 450 nm and 4.56 mW cm^−2^ light, the coupled nanogenerator achieved a *E*_max_ ~ 6.13%, with the transferred charge being 40.14, 42.64 and 0.036 μC for the photoelectric, coupled and flexoelectric effects, respectively. As a result of the small values, the flexoelectric signal is susceptible to environmental influences, but the induced transfer charge is far less than the photoelectric and coupling effects; therefore, the errors caused by flexoelectric can be ignored when calculating the degree of charge enhancement. For better exploration of harvesting multiple sources to study the enhanced coupling performance, Fig. 4b clearly shows the difference in the electrical signal of the nanogenerator under vibration alone, light alone, and combined vibration and light states in terms of five key parameters: current, voltage, charge, energy and power. This demonstrates in multiple dimensions the effective modulation of the photoelectric effect by the flexoelectric effect, and the coupling enhancement achieved by the coupled nanogenerator in several aspects. To evaluate the effectiveness of coupling, the Yang coupling factor of the device is defined as *K*_*C,A*_:

2 $$K_{C, \, A} = B/\Sigma \, b_{i}$$

*A* is one of current, voltage, charge, energy, and power. *B* is the signal value after coupling and *b*_*i*_ is the signal value for the various states prior to coupling. The Yang coupling factors for charge and energy at different wavelengths are shown in Fig. 4c, d. The Yang coupling factors for the three independent cases are compared in Fig. 4c, and the Yang coupling factors are affected by a wide range of variations in light intensity. In addition, we observe the Yang coupling factors of various physical device quantities (charge, energy, current, voltage and power) vary at the same operating conditions (Fig. 4e), and the Yang coupling factors of the other physical quantities at different wavelengths are shown in Fig. S16. The Yang coupling factor for the current is the largest, and the Yang coupling factor of the charge obtained by integrating the current is significantly smaller; this is due to the coupling current varying from moment to moment during vibration, and the instantaneous coupling-enhanced current is used in the calculation of the current coupling factor.

Due to the combination of the light-induced heating thermal effect and an opposing cooling effect induced by the vibration of the film, the current generated by the coupling effect at high light intensities is smaller than that corresponding to the linear curve show Fig. 3b; therefore, a small range of light intensities of 1–10 4.5 mW cm^−2^ is chosen to be further optimized from the wide range (Fig. S4).

### Mechanism of Operation of the Coupled Device

**Fig. 5:**
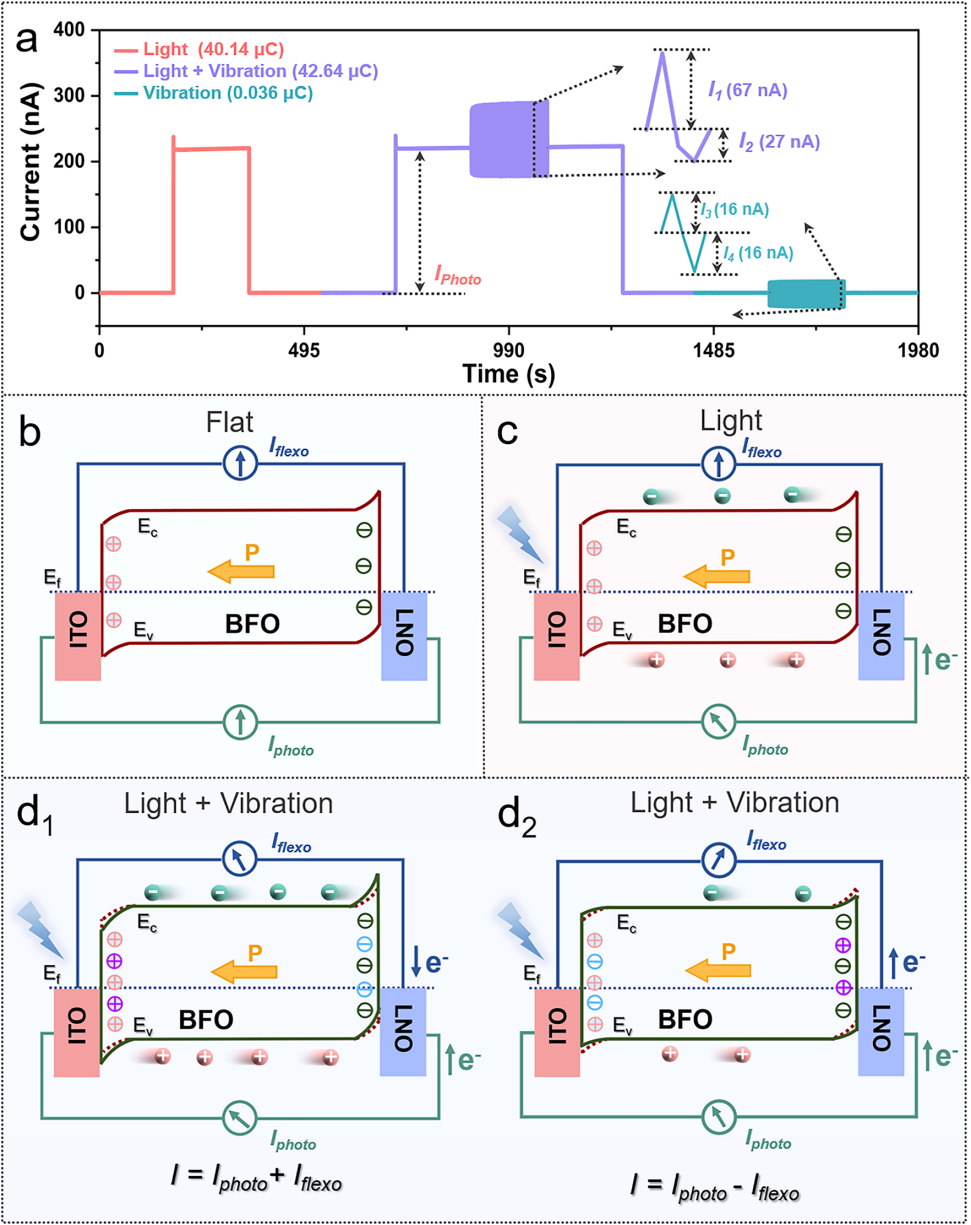
Working principle of LNO/BFO/ITO coupled nanogenerators. **a** Representative output curve of the device with simultaneous light and vibration operation. Mechanisms of device operation at **b** flat, **c** light, **d** light and vibration

Representative curves for light and vibration applied individually or simultaneously to the LNO/BFO/ITO device structure are shown in Fig. 5a, corresponding to output currents of 221, 288 and 16 nA, respectively. Comparing the signal amplification plots of the device under combined light and vibration and vibration alone combined light and vibration and vibration alone, the absolute values of the AC signals corresponding to the flexoelectric effect are essentially equal, while the absolute value of the difference between the photoelectric-flexoelectric signal and the photoelectric signal differs by a factor of 2.48, which is an important indication of the enhanced coupling of the device.

To further determine the photoelectric-flexoelectric coupling effect and to exclude the influence of the distance of the light source from the device while it is vibrating, we tested the output signal at 450 nm for different vibration modes (Fig. S17), as the specific test details are in the supplemental data manuscript. We observed that the device reached a maximum charge enhancement ratio *E* of 6.13% in the tested vibrational mode, which is significantly larger than the other vibrational modes, proving the effective modulation of the photoelectric effect achieved by dynamic flexoelectric effects.

In this work, the BFO ferroelectric film output signal is influenced by both the internal polarization-induced electric field *E*_*in*_ and the electric field *E*_flexo_ induced by the flexoelectric effect. As a result, carriers within the ferroelectric are efficiently separated and transported by the combined action of two electric fields [22, 30], while the flexoelectric and photovoltaic signals exhibit AC and DC features, respectively, which are labelled in separate circuits to distinguish them. The energy level diagrams for each constituent material of the device are shown in Fig. S19a [36, 37]. The ideal diagram for the LNO/ITO/BFO junction, without considering the ferroelectric polarization, is shown in Fig. S19b. Due to the ferroelectric spontaneous polarization (*P*) inside the BFO ferroelectric film with LNO pointing toward the ITO, the positive (negative) charges accumulated cause the potential barrier between the ferroelectric film and metals to become smaller (larger) and can reverse the initial energy band bending (Fig. 5b) [38]. Under the application of light alone (450 nm), electrons and holes are separated and transferred by the *E*_in_, with the external current of LNO to ITO, where there is no flow of electrons in the flexoelectric circuit (Fig. 5c). The enhanced performance of the photoelectric-flexoelectric coupling can be illustrated by the flexoelectric effect affecting the energy band bending of the BFO. On one hand, when the device is flexed, the redistributed negative charges at the BFO/LNO raise the BFO band energy level, and the built-in electric field becomes stronger; thus, the photovoltaic performance is enhanced. On another hand, the *E*_*flexo*_ drives charge redistribution in the BFO film, generating a current from the LNO to the ITO, which is in the same direction as the photocurrent in the flexoelectric circuit; therefore, the instantaneous output signal is improved (31%) (Fig. 5d). The current flow and magnitude when the device is bent in the opposite direction are shown in Fig. 5e. Here, the output current of the coupling effect is reduced (by 12%) because the flexoelectric effect causes the potential barriers to change in the opposite direction, with a weaker built-in electric field and electrons flowing in the bending circuit in the opposite direction to the photocurrent.

### Charging Effect of the Coupled Device

After determining the coupling enhancement of the coupled nanogenerator based on the BFO ferroelectric film, we further verified the effective simultaneous harvesting of vibration and light energy, and the energy harvesting and measured voltage curves of capacitors under different working conditions are shown in Fig. S20. Due to the AC characteristics of the flexoelectric signal, energy harvesting by the BFO coupled nanogenerator is not achieved when vibrating alone, corresponding to a negligible amount of charge migration (0.036 µC) in Fig. 4a. At a light intensity of 4.5 mW cm^−2^, the photoelectric-flexoelectric effect of the device corresponds to a significantly superior energy harvesting and charging capacity compared to that of the photoelectric effect alone, regardless of the wavelength of light, successfully realizing the effective harvesting of light and vibration energies. The representative curve of the voltage output (Fig. S20d) also demonstrates that the signal under "light and vibration" (105 mV) is significantly larger than under "light" (91 mV). Furthermore, the measured voltage curves of the nanogenerator for different capacitors under the same illumination at the same time and the curves for the same capacitor (47 μF) under different wavelengths of light at the same time are shown in Figs. S20e and S24, demonstrating that the photoelectric-flexoelectric effect successfully leads to the increase in collected charges and the effective collection of vibration energy and that the realism and reproducibility of performance improvements of coupled nanogenerators.

Finally, we investigated the durability and stability characteristics of the coupled nanogenerator, as shown in Fig. S26, which are key requirements for the application. The current signals of photoelectric, flexoelectric and photoelectric-flexoelectric effects of the nanogenerator are continuously detected at the frequency of 17 Hz, and negligible attenuation of the signals can be observed for eight 360 s periods. The excellent stability and reliability offer more possibilities for the long-term use of the devices while providing an efficient way of harvesting solar and wind energy that can be found everywhere in the environment.

## Conclusion

We have constructed a new form of low-cost and environmentally friendly coupled nanogenerator based on a flexible lead free BiFeO_3_ (BFO) ferroelectric thin film to harvest both light and vibration energies. By careful introduction of strain gradients via mechanical vibrations, the coupled nanogenerator couples both photoelectric and flexoelectric effects to achieve enhancements in multiple performance parameters such as current, voltage, charge, energy and power, to successfully harvest light and vibration energy simultaneously. A detailed evaluation of the device response under a range of conditions of light and vibration has led to new insights into the coupling mechanisms and enhanced performance. Effective regulation of the electrical charge is achieved by the combined effect of the electric field formed by flexoelectric effect and the built-in electric field associated with the ferroelectric polarization of the BFO film. As a result, the output signal of the device is effectively improved by up to 131% under low light conditions. Our research provides a new strategy for scavenging a variety of forms of ambient energies and the creation of self-powered multifunctional harvesting. The sensor devices that couple multiple harvesting mechanisms and exploit the intriguing multifunctional properties of flexible ferroelectric films are prepared as new functional devices.

## Supplementary Information

Below is the link to the electronic supplementary material.

Supplementary file1 (MP4 12765 kb)

Supplementary file2 (PDF 5020 kb)
