# Supplementary material for: Coupling Enhancement of a Flexible BiFeO3 Film-Based Nanogenerator for Simultaneously Scavenging Light and Vibration Energies
Source: Nanomicro Lett. 2022 Oct 6;14:198. doi: 10.1007/s40820-022-00943-0 (PMC9537400; doi:10.1007/s40820-022-00943-0)
Supplement: Supplementary file 2 — Supplementary file2 (PDF 5020 kb) [file 40820_2022_943_MOESM2_ESM.pdf]

Supporting Information for

Coupling Enhancement of a Flexible $BiFeO_3$ Film-Based Nanogenerator for Simultaneously Scavenging Light and Vibration Energies

Xiao Han[1, 3], Yun Ji[1, 3], Li Wu[1, 4], Yanlong Xia[1, 4], Chris R. Bowen[2], and Ya Yang[1, 3, 4, *]

[1] CAS Center for Excellence in Nanoscience, Beijing Key Laboratory of Micro-nano Energy and Sensor, Beijing Institute of Nanoenergy and Nanosystems, Chinese Academy of Sciences, Beijing 101400, P. R. China

[2] Department of Mechanical Engineering, University of Bath, BA27AK, UK

[3] School of Nanoscience and Technology, University of Chinese Academy of Sciences, Beijing 100049, P. R. China

[4] Center on Nanoenergy Research, School of Physical Science and Technology, Guangxi University, Nanning 530004, P. R. China

*Corresponding author. E-mail: yayang@binn.cas.cn (Ya Yang)

Supplementary Figures

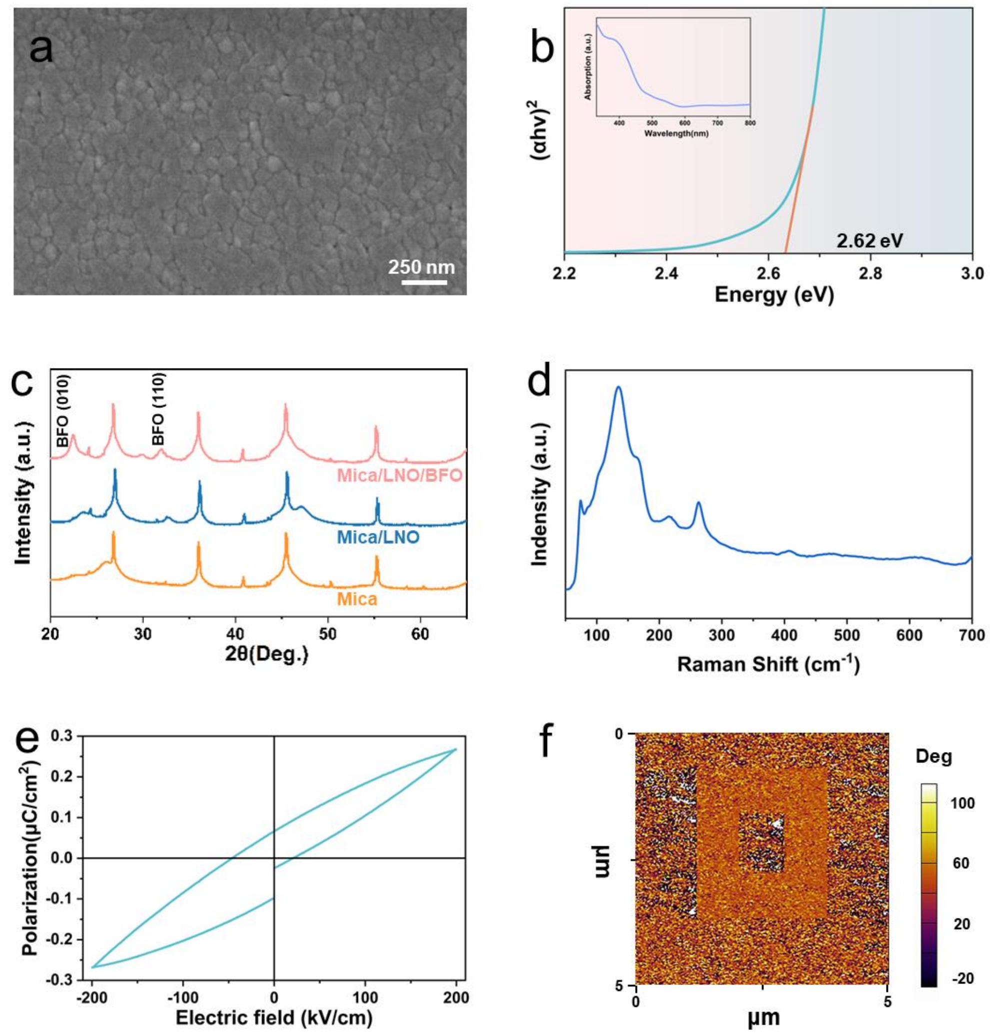

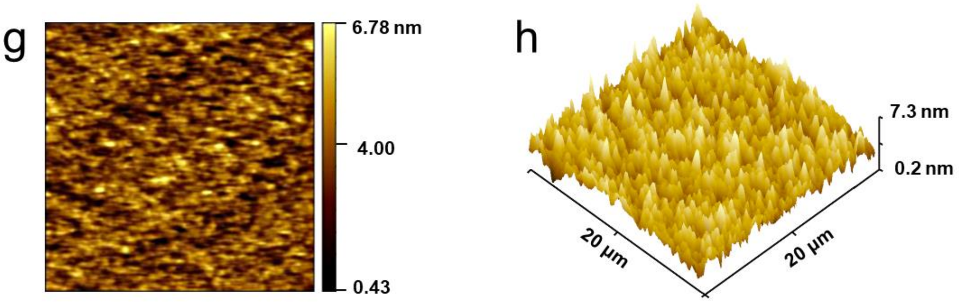

Fig. S1 Material characterization of BFO ferroelectric films. (**a**) SEM image of BFO surface morphology. (**b**) Absorption spectrum of BFO films. (**c**) XRD comparison of the different components of the device. (**d**) The Raman spectrum of BFO films. (**e**) P-E loop for BFO (electrode area 1 x 1 mm^2). (**f)** PFM phase images of the BFO film, with -2 and +2 V bias for the virgin and switched states respectively. (**g-h**) AFM images of BFO films

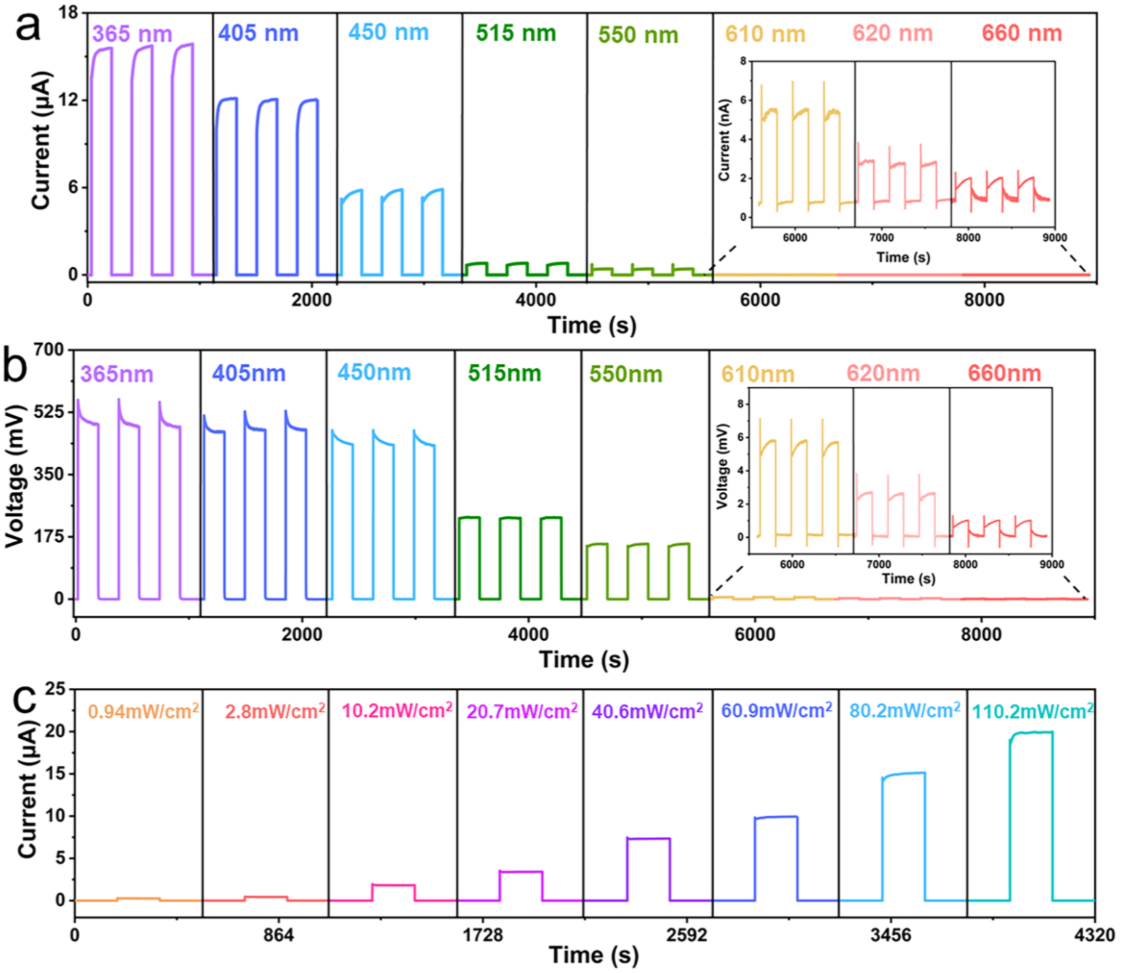

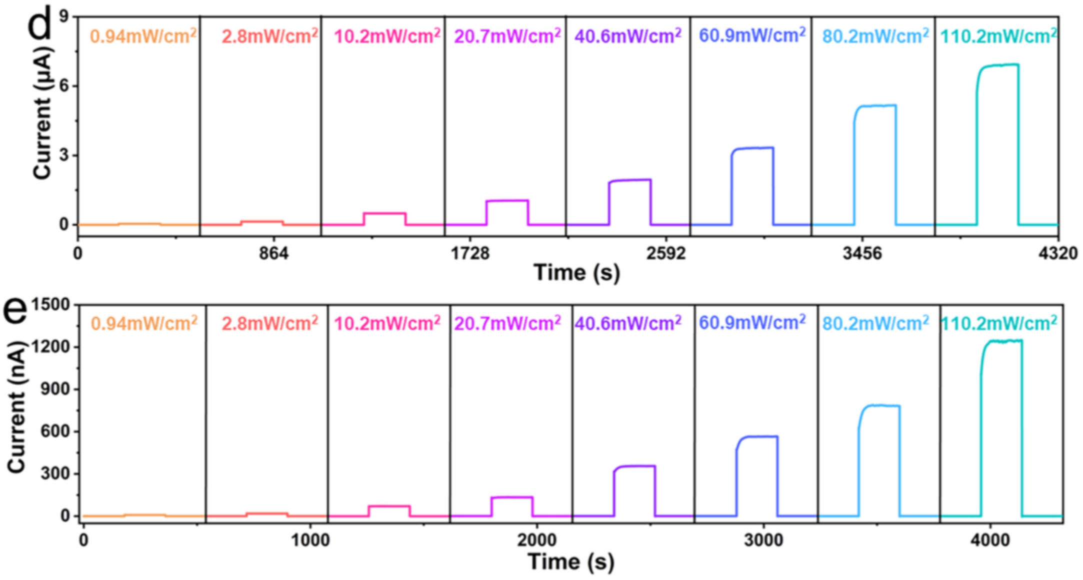

Fig. S2 Photovoltaic signals for devices. (**a-b**) Short-circuit current and open-circuit voltage of nanogenerators under different wavelengths of light illumination (75 mW/cm^2). Short-circuit current of the device under light illumination of different light intensities at (**c**) 365 nm, (**d**) 450 nm and (**e**) 515 nm

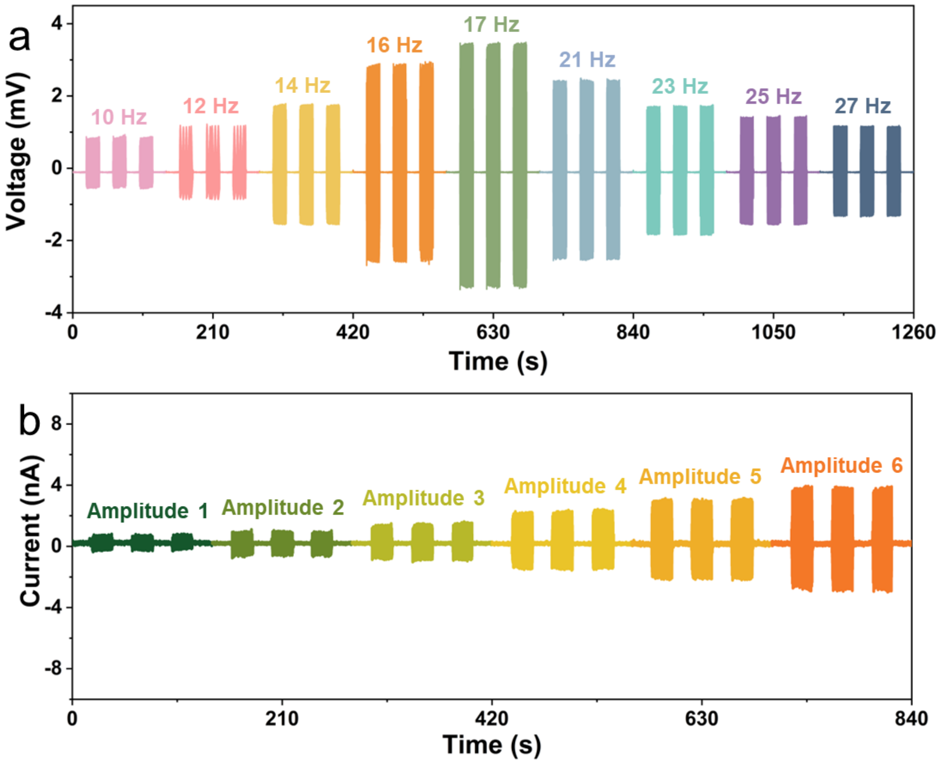

Fig. S3 Flexoelectric signals for devices. (**a**) Flexoelectric voltage signals with different frequency. (**b**) Flexoelectric current signals at different amplitudes (10 Hz)

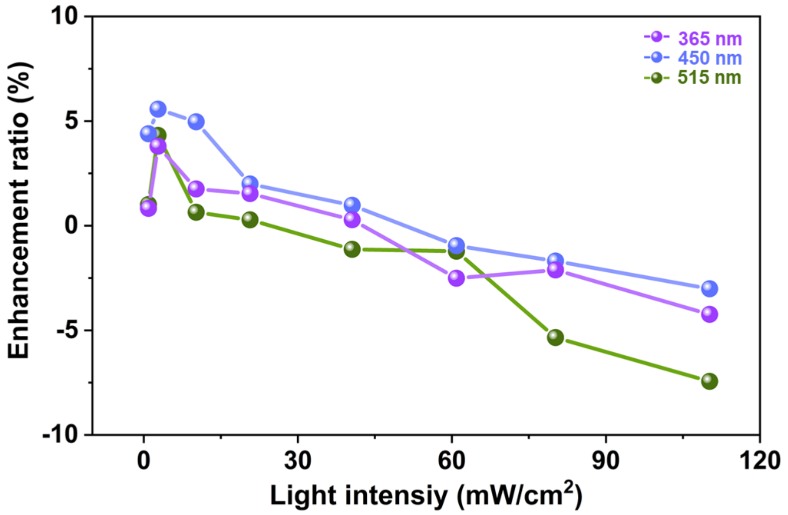

Fig. S4 Charge enhancement ratio E as a function of light intensity for three wavelengths over a wide range of light intensities. Due to the combination of the light-induced thermal effect and the cooling effect caused by vibration, the current of the coupling effect at large light intensities is smaller than that corresponding to the linear curve, and the collected charges all show experimental results of “1+1<2”. Considering that the device differs in several factors such as the separation ability of photogenerated carriers and temperature variations under different circumstances, the range of charge enhancement above 0 is different for different wavelengths, so the small range of light intensities of 1-10 is chosen to be further optimised from the wide range

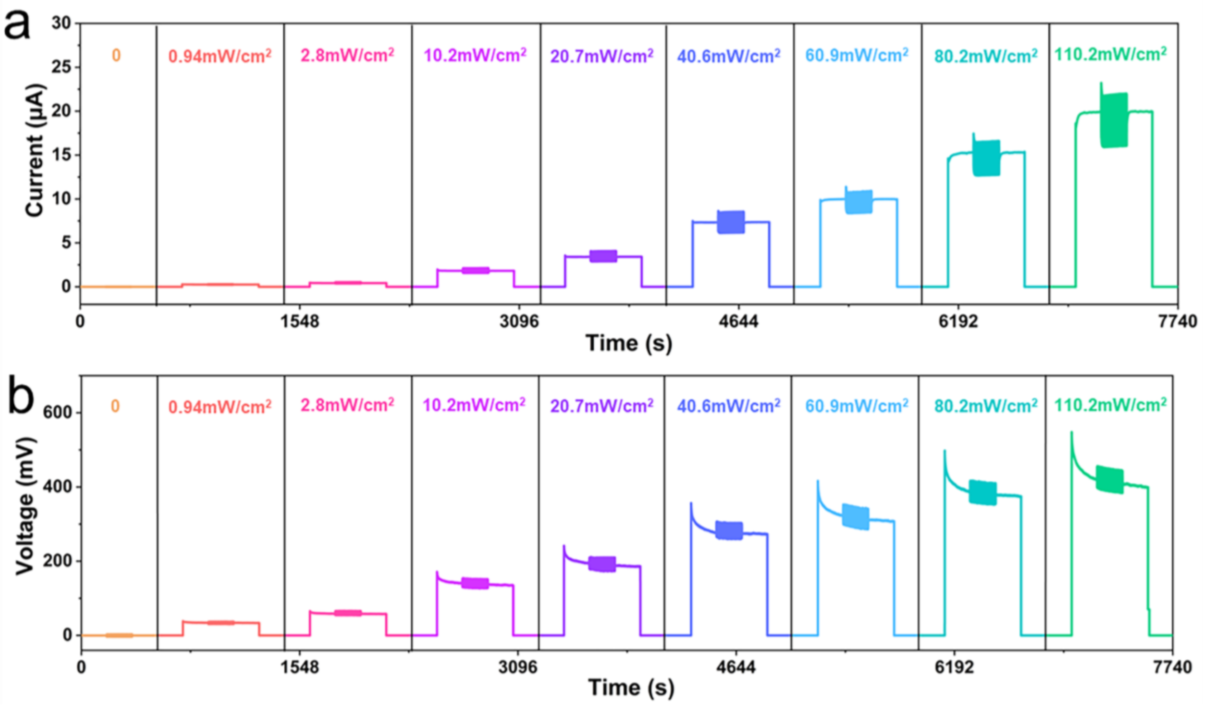

Fig. S5 The effect of light-vibration coupling on the output current and output voltage of nanogenerators at 365 nm with different light intensities

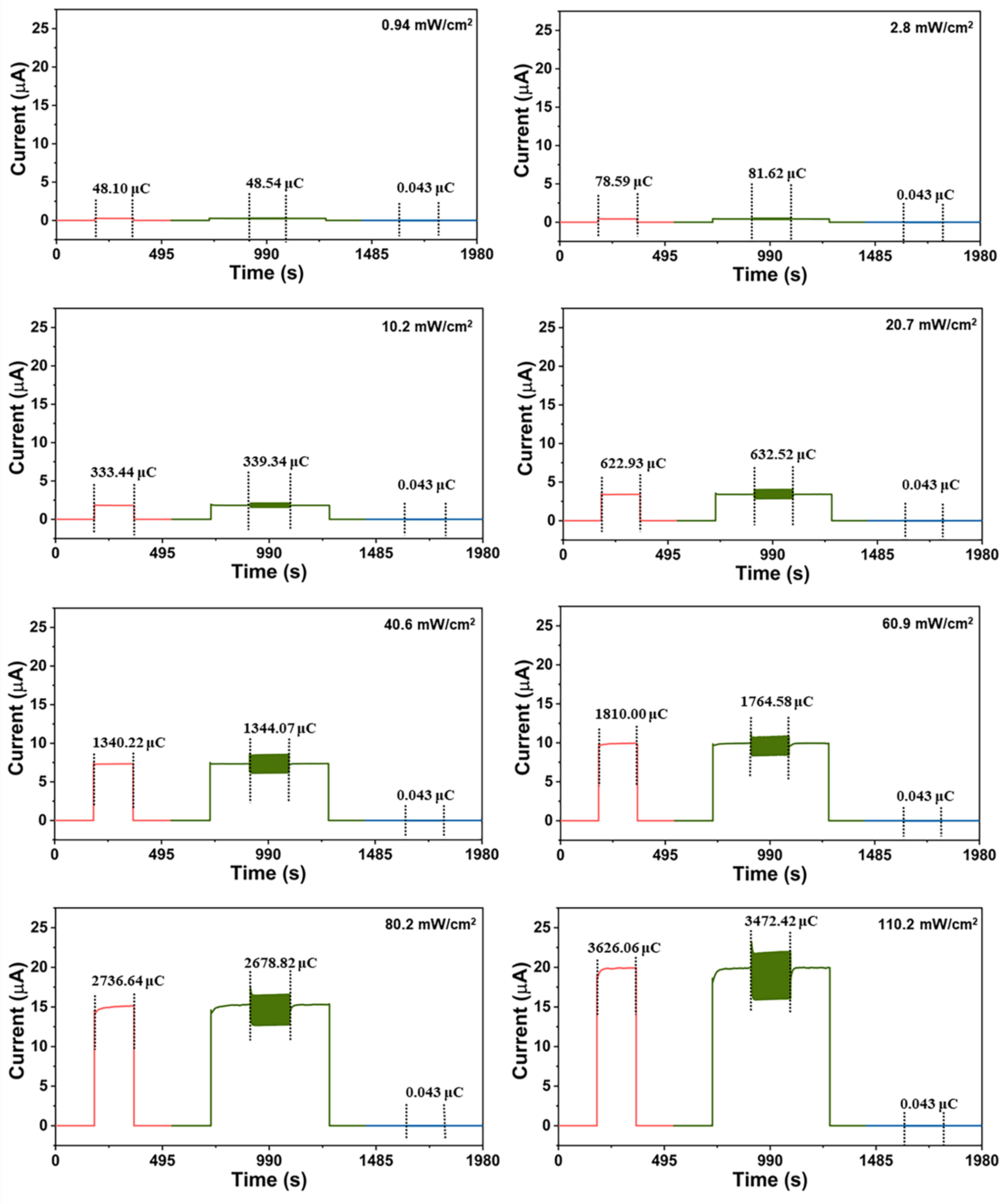

Fig. S6 Light, vibration, and light followed by vibration on output current of the nanogenerator under different light intensities at 365 nm (0.94 - 110.2 mW/cm^2)

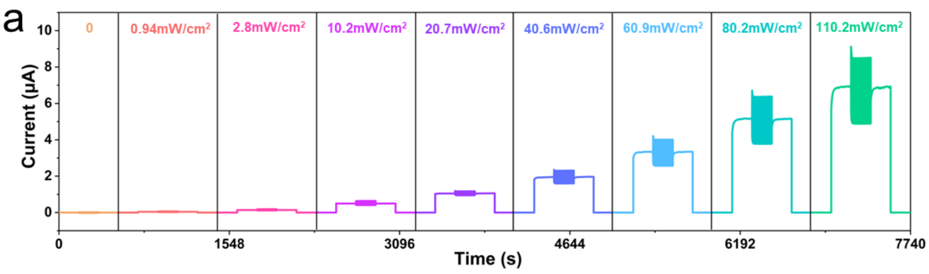

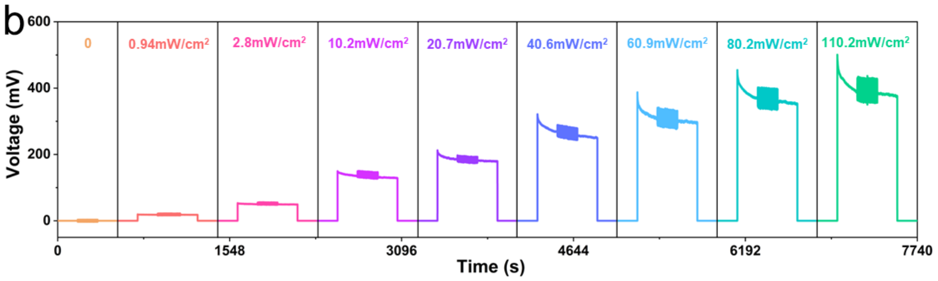

Fig. S7 The effect of light-vibration coupling on the output current and output voltage of nanogenerators at 450 nm with different light intensities

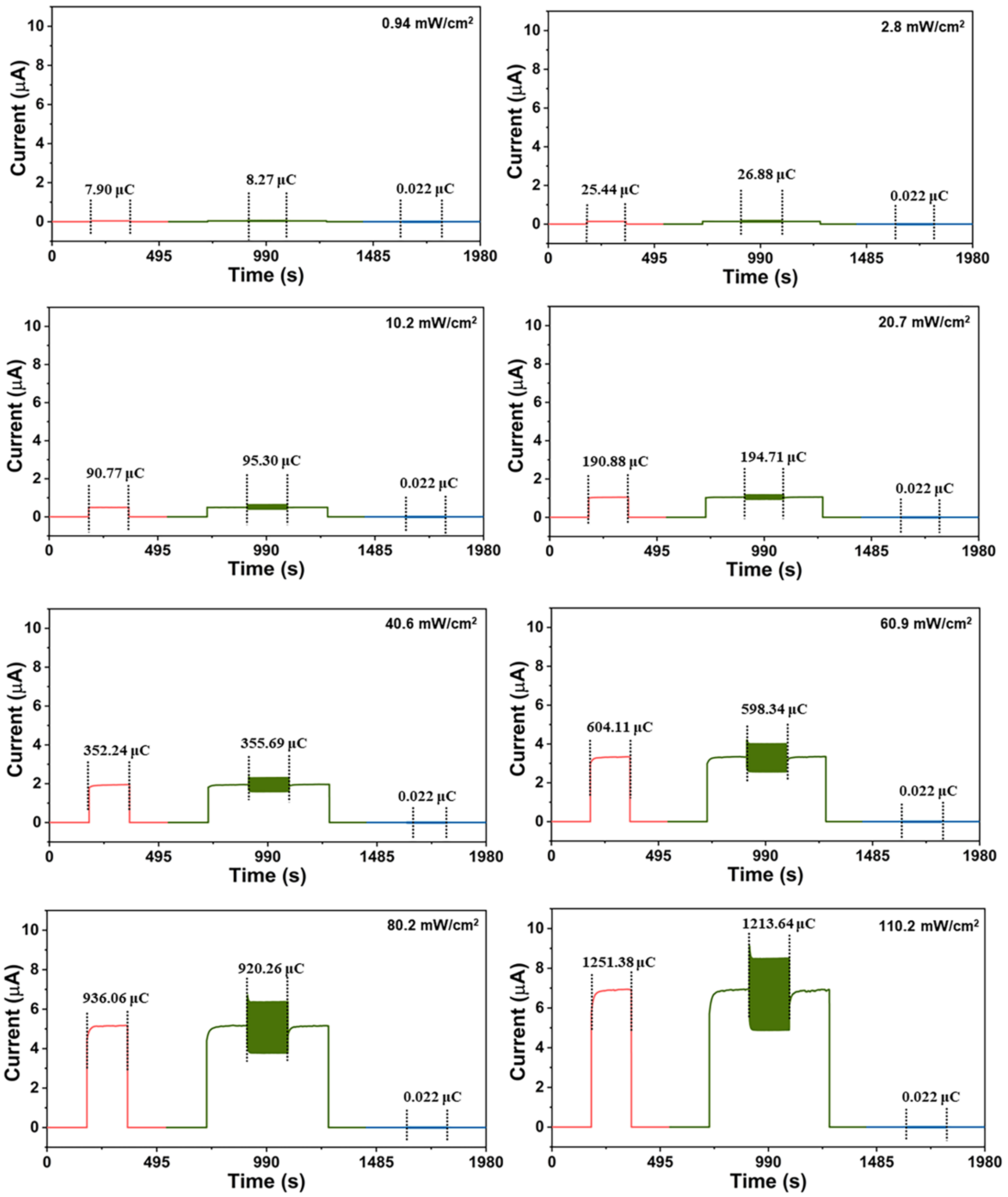

Fig. S8 Light, vibration, and light followed by vibration on output current of the nanogenerator under different light intensities at 450 nm (0.94 - 110.2 mW/cm^2)

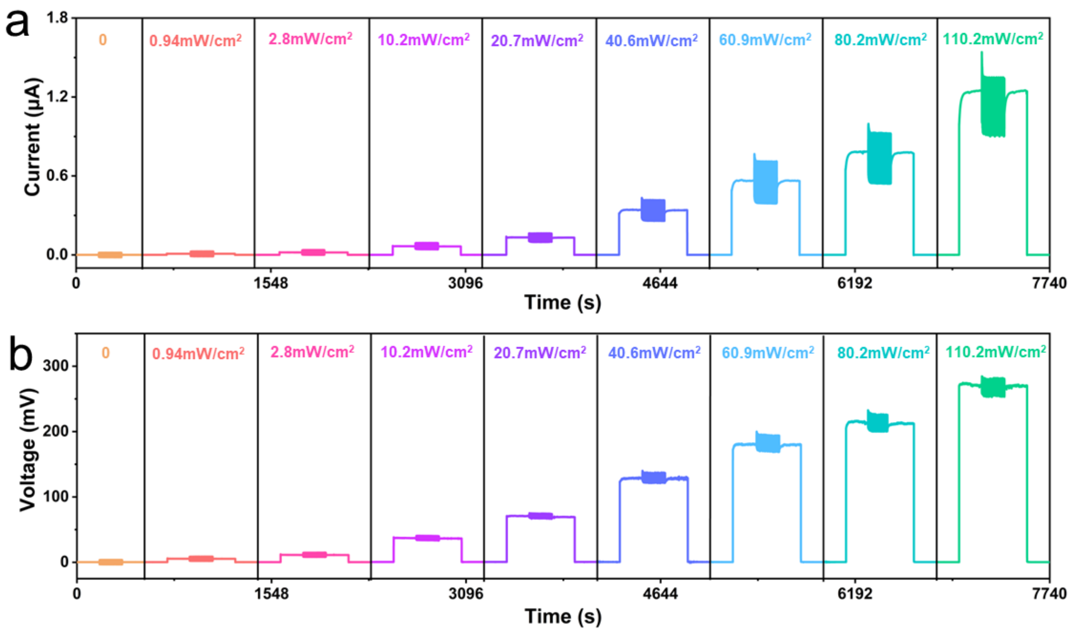

Fig. S9 The effect of light-vibration coupling on the output current and output voltage of nanogenerators at 515 nm with different light intensities

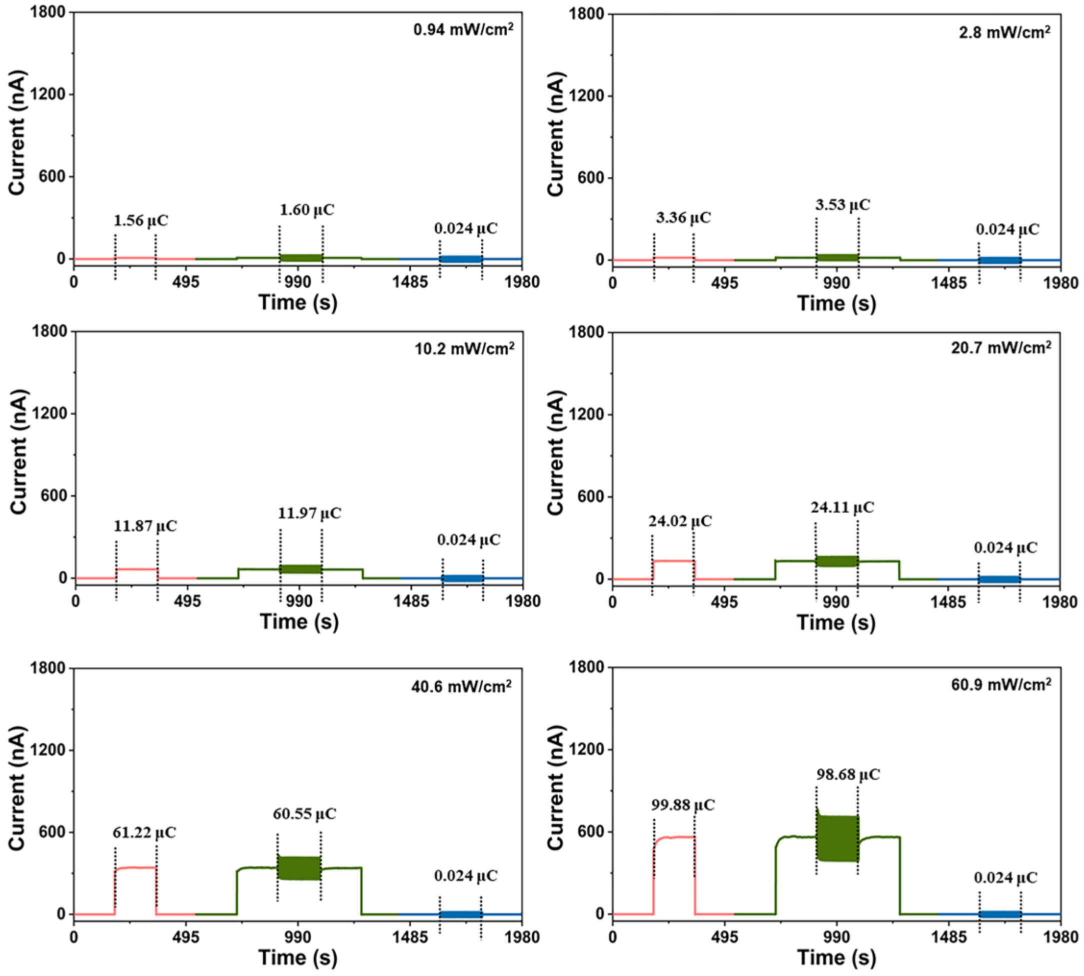

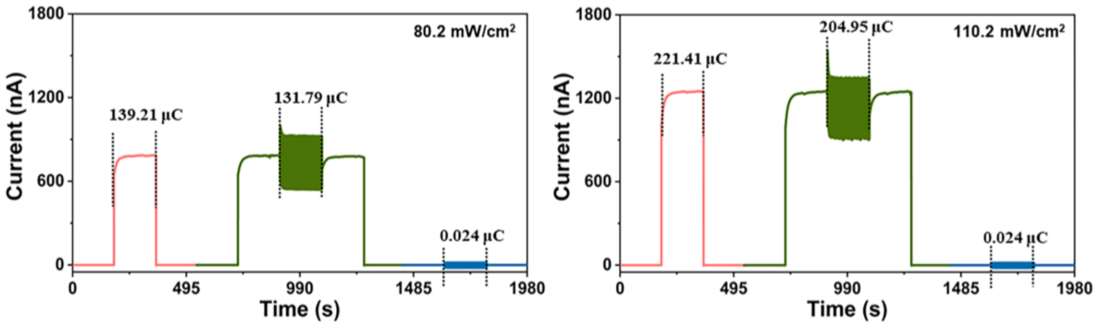

Fig. S10 Light, vibration, and light followed by vibration on output current of the nanogenerator under different light intensities at 515 nm (0.94 - 110.2 mW/cm^2)

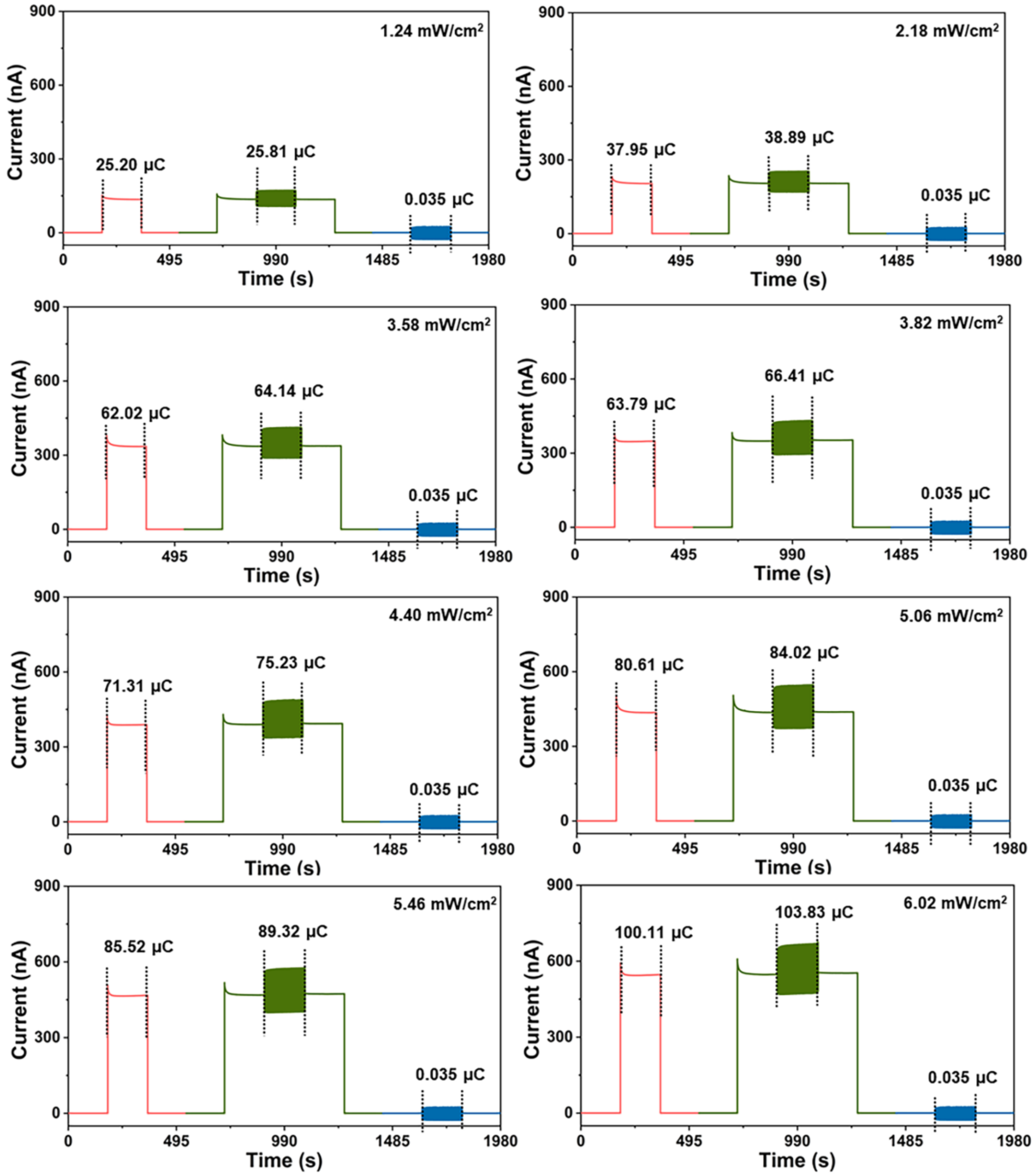

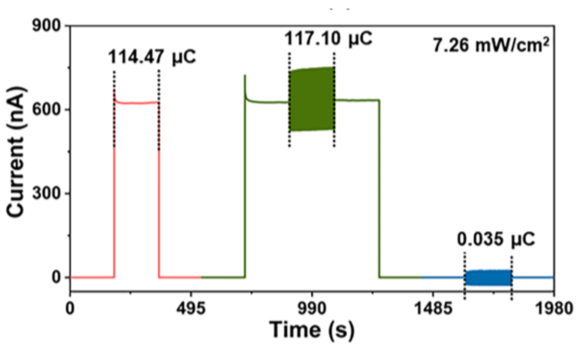

Fig. S11 Light, vibration, and light followed by vibration on output current of the nanogenerator under different light intensities at 365 nm (1- 7 mW/cm^2)

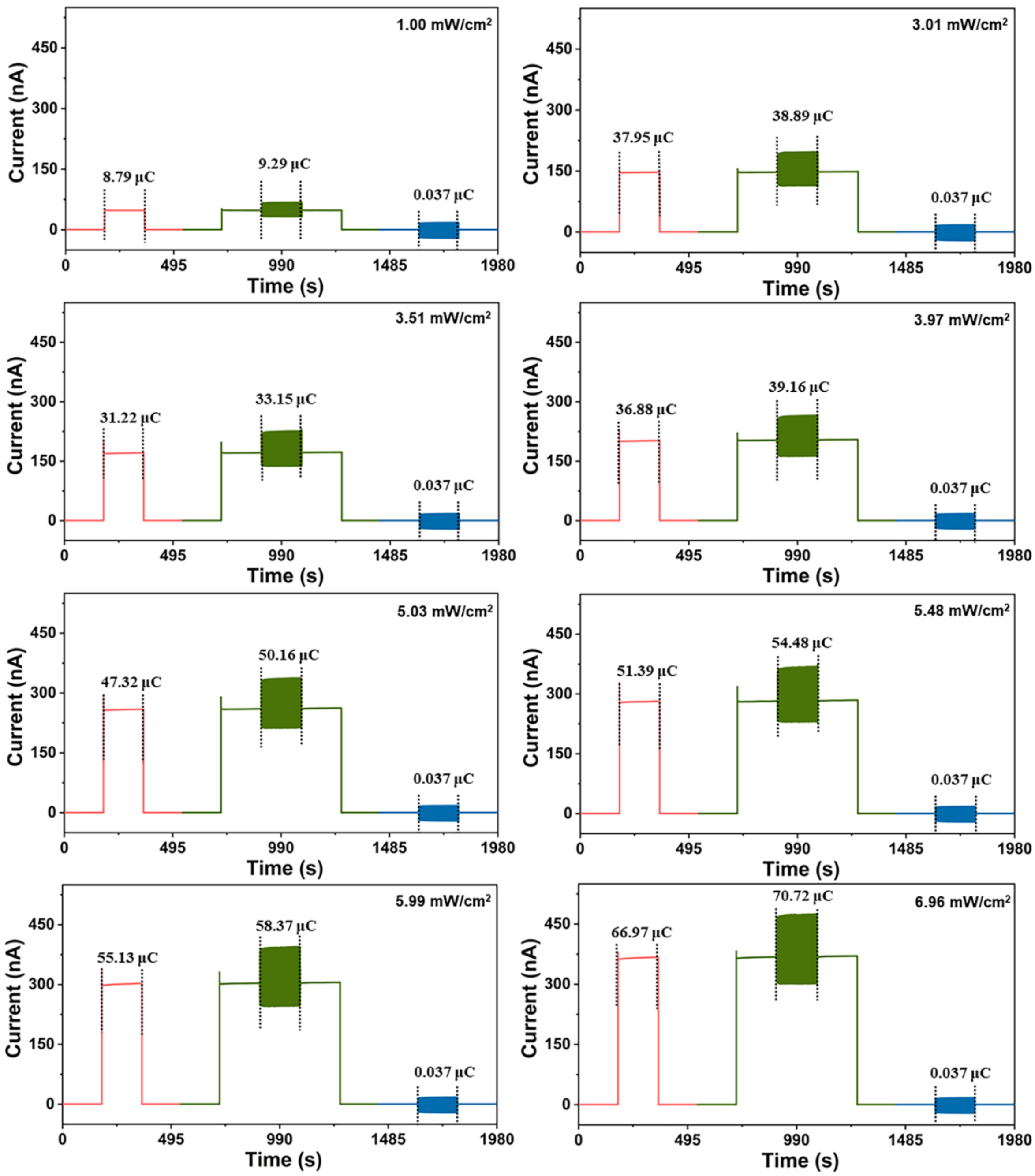

Fig. S12 Light, vibration, and light followed by vibration on output current of the nanogenerator under different light intensities at 450 nm (1- 7 mW/cm^2)

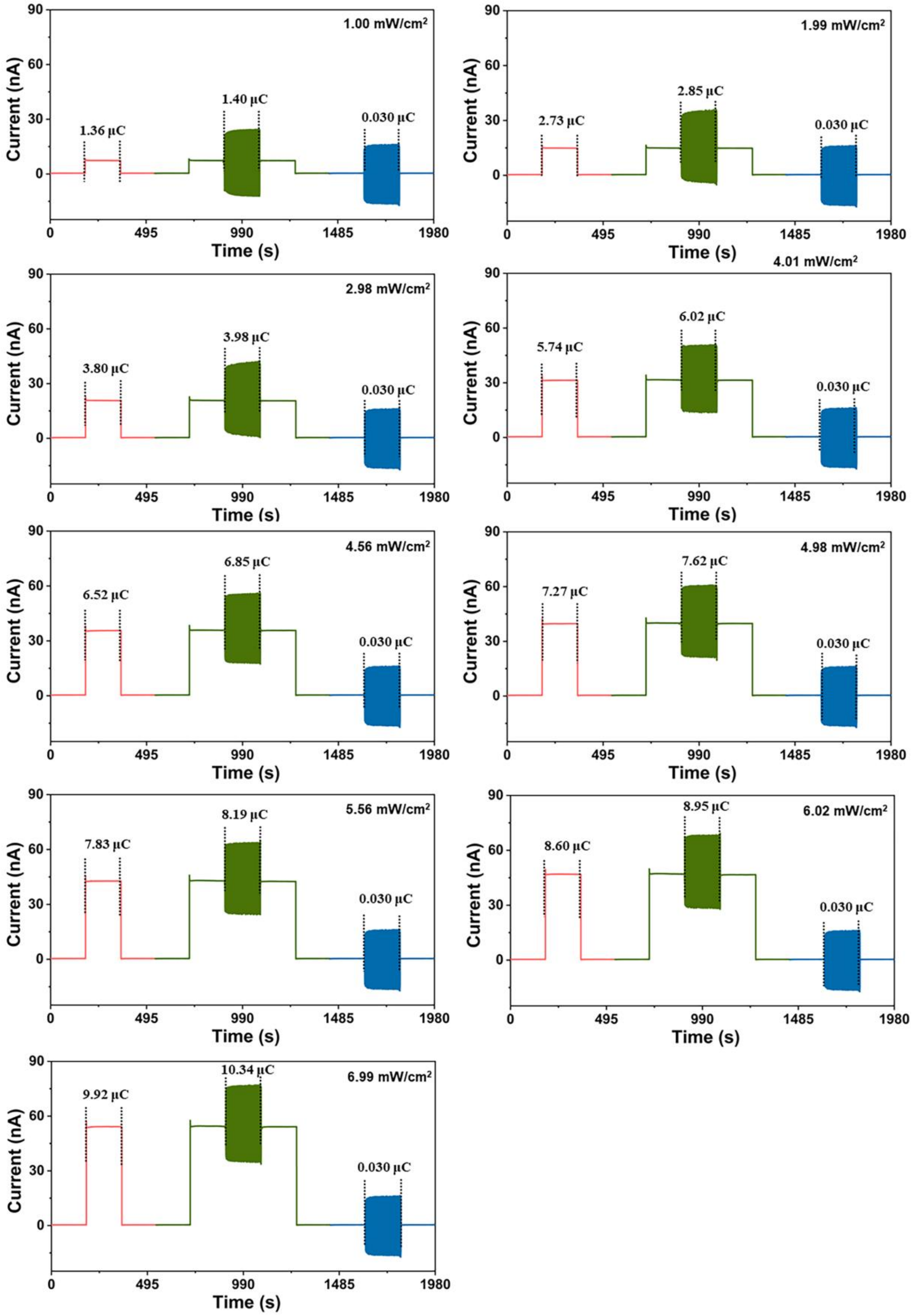

Fig. S13 Light, vibration, and light followed by vibration on output current of the nanogenerator under different light intensities at 515 nm (1- 7 mW/cm^2)

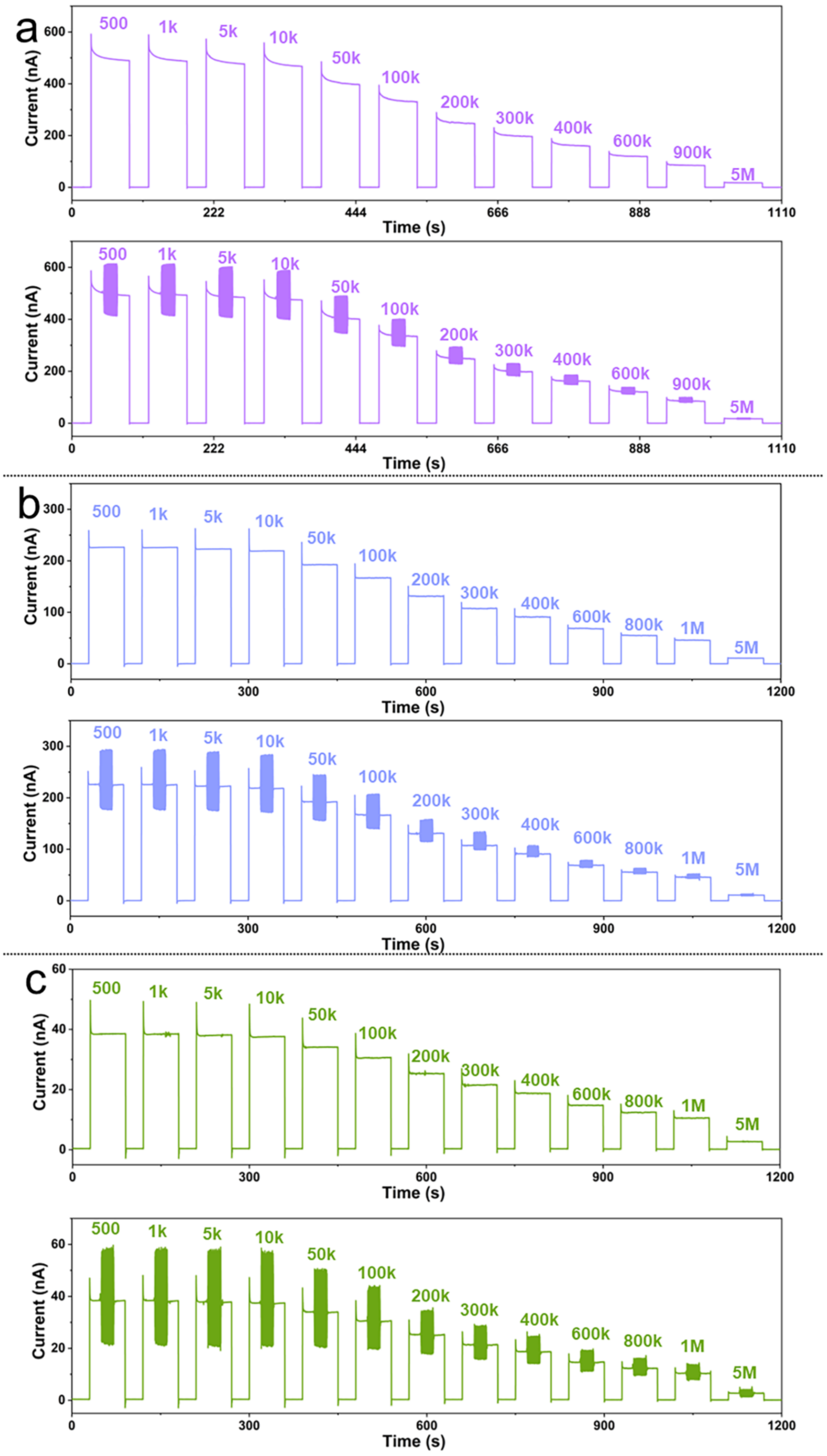

Fig. S14 Load-dependent photovoltaic, photoelectric-flexoelectric output current for devices at (**a**) 365 nm, (**b**) 450 nm and (**c**) 515 nm (4.5 mW/cm^2)

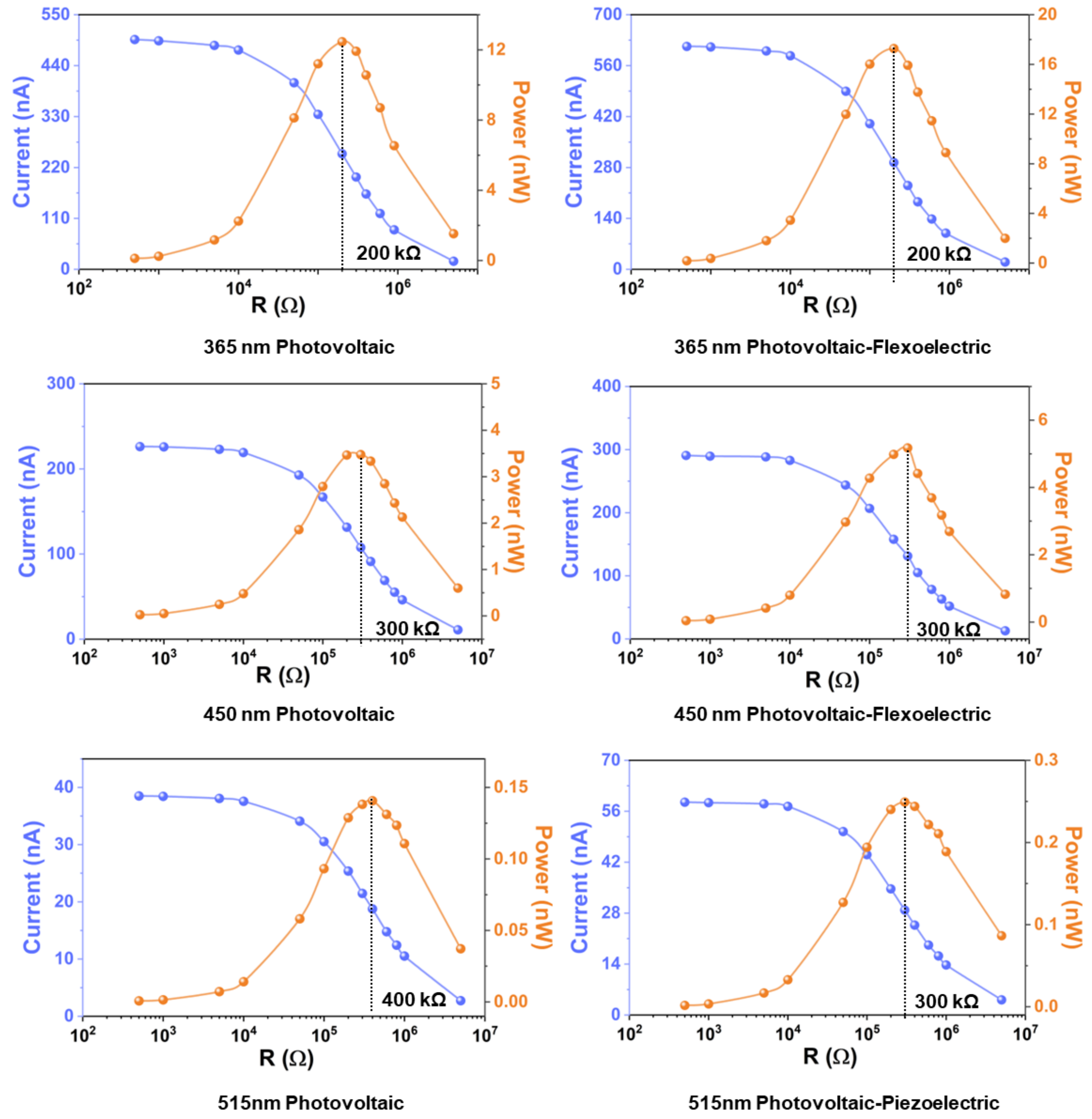

Fig. S15 Load-dependent output current and power of the nanogenerator relating to the photovoltaic effect and photovoltaic–flexoelectric coupling effect (4.5 mW/cm^2)

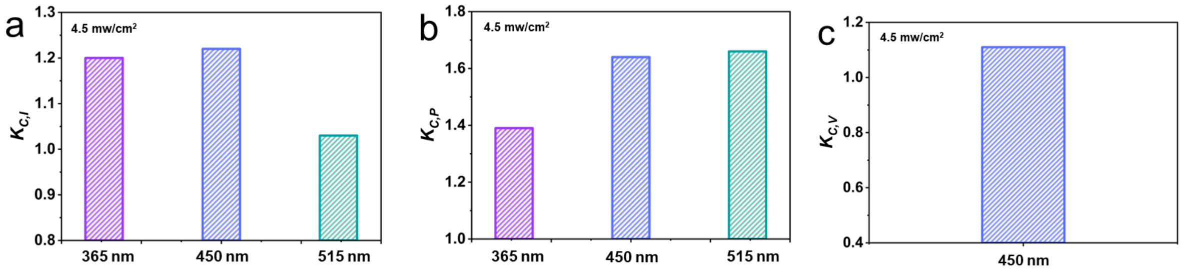

Fig. S16 Coupling coefficients of coupled nanogenerators. (**a**) Current coupling coefficient $K_{C,I}$. (**b**) Power coupling coefficient $K_{C,P}$. (**c**) Voltage coupling coefficient $K_{C,V}$

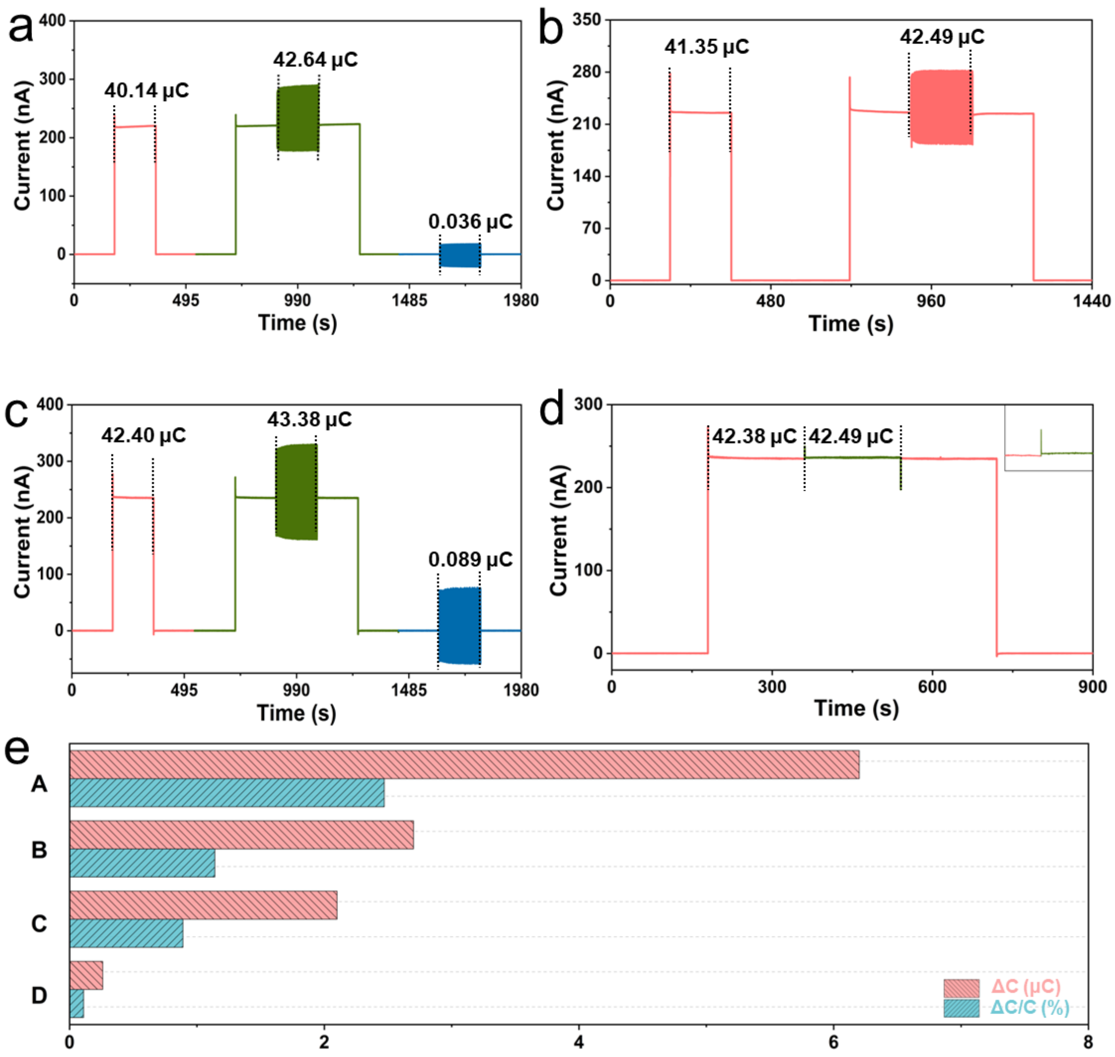

Fig. S17 Comparative tests. (**a-d**) Current output signals for different vibration conditions under 450 nm illumination. (**e**) Comparison diagram of charge difference for different vibration modes ($\Delta C = C_{photo\text{-}flexo} - C_{photo}$)

Mode A is the test mode, where strain gradients are introduced by the cantilever beam structure and vibration to produce flexoelectric signals, while mode B and C is the comparison test without the influence of the light source. Mode B uses a device that remains flat and moves up and down with the vibration exciter, where the distance of the light source plays an absolute role. Mode C also excludes the influence of the light source, the device is not moved with the vibration exciter, but the film is bent by the impact of the object on the vibration exciter, which introduces strain gradients. Even though mode C uses the conditions of 17 Hz and minimum amplitude, the corresponding bending radius is larger than mode A due to the impact of the object, which corresponds to a larger electrical signal of flexoelectric effect. However, under the same frequency and large amplitude, the mode C loses its regularity of vibration and loses its potential for application in more situations. Mode D indicates the coupling of light and vibration at rest. Excluding the influence of the light source, the coupling effect at rest is tested and the device undergoes a small deformation, corresponding to the generation of a current signal. The results show that the largest charge enhancement ratio E (6.13%) is achieved when the device is vibrating up and down with the vibration exciter and bending itself, and that the result for charge enhancement ratio in mode C (2.10%) is less than the difference (3.37%) between A and B (2.76%).

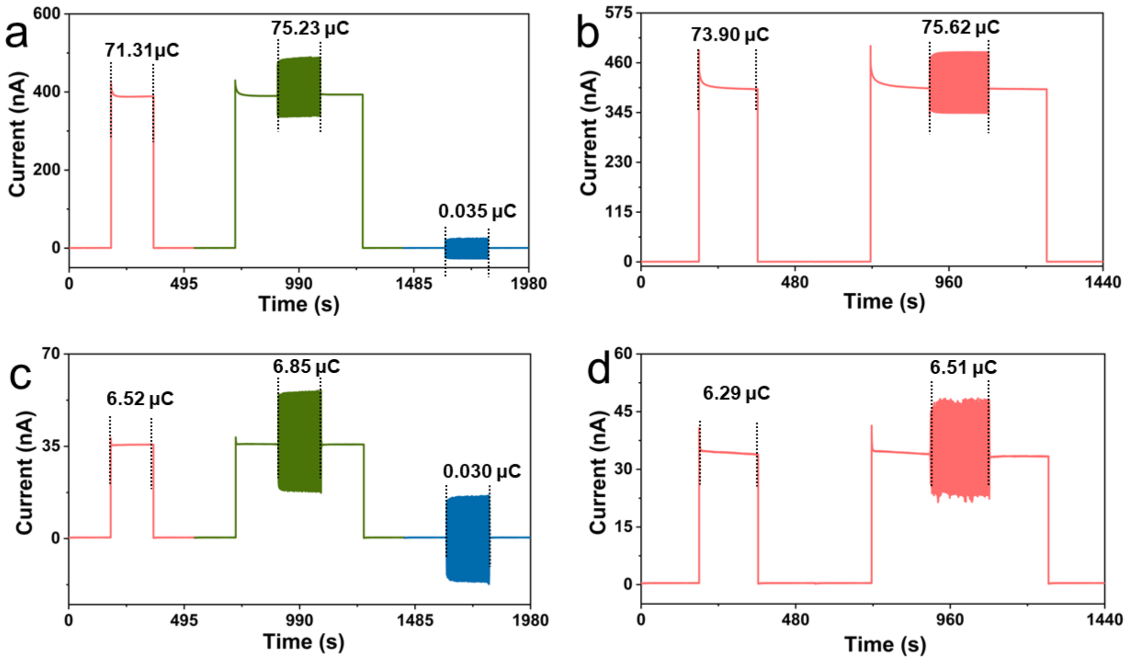

Fig. S18 Comparative tests. Current output signals for different vibration conditions under (**a-b**) 365 nm illumination and (**c-d**) 515 nm illumination

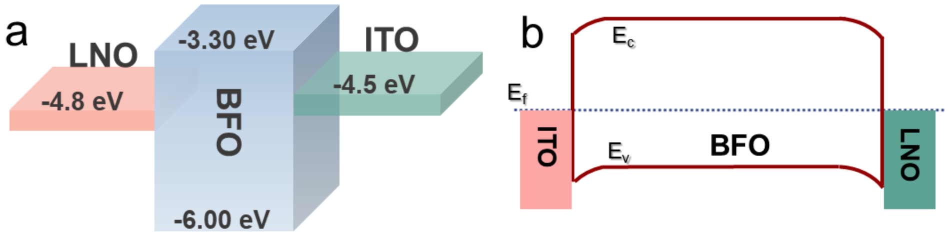

c

Sample	Hall coefficient (cm³/C)
Mica/LNO	-1.142E-03 -2.413E-03 -6.747E-03
Mica/LNO/BFO	2.083E-03 3.339E-02 6.209E-02

Fig. S19 (**a**) Energy level diagram for each material of the device. (**b**) Energy band bending of devices without consideration of spontaneous polarization. (**c**) Hall coefficients of the individual components of the coupled nanogenerators

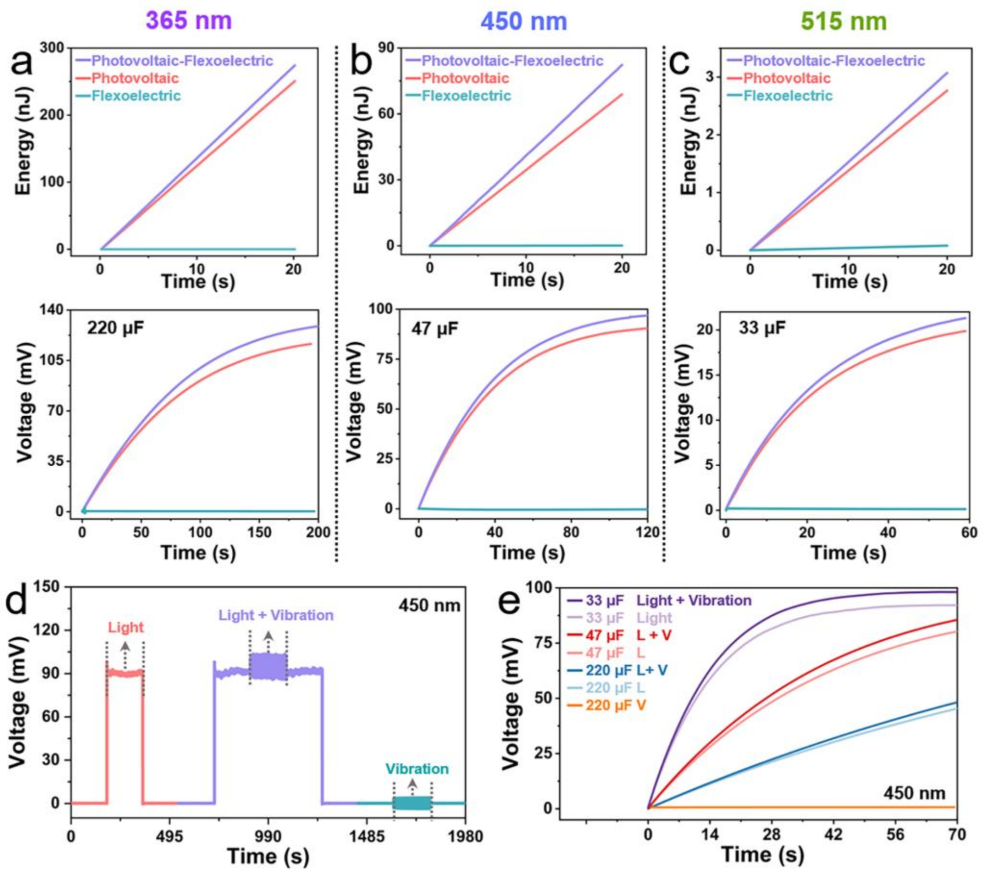

Fig. S20 Charging performance of LNO/BFO/ITO coupled nanogenerators. Energy harvesting curves and measured voltage curves for charging at (**a**) 365 nm (**b**) 450 nm and (**c**) 515 nm (4.5 mW/cm^2). (**d**) The voltage output curve of the device when operating simultaneously with light and vibration. (**e**) Measured voltage curves for the charging of different capacitors, corresponding to the state of d

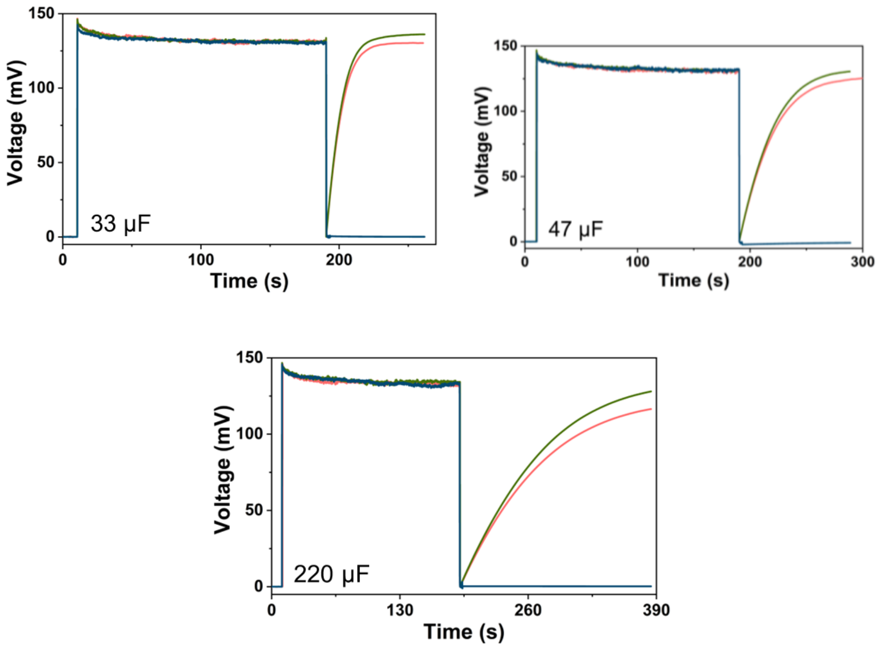

Fig. S21 The measured voltage curves of different capacitors under 365 nm light illumination (4.5 mW/cm^2)

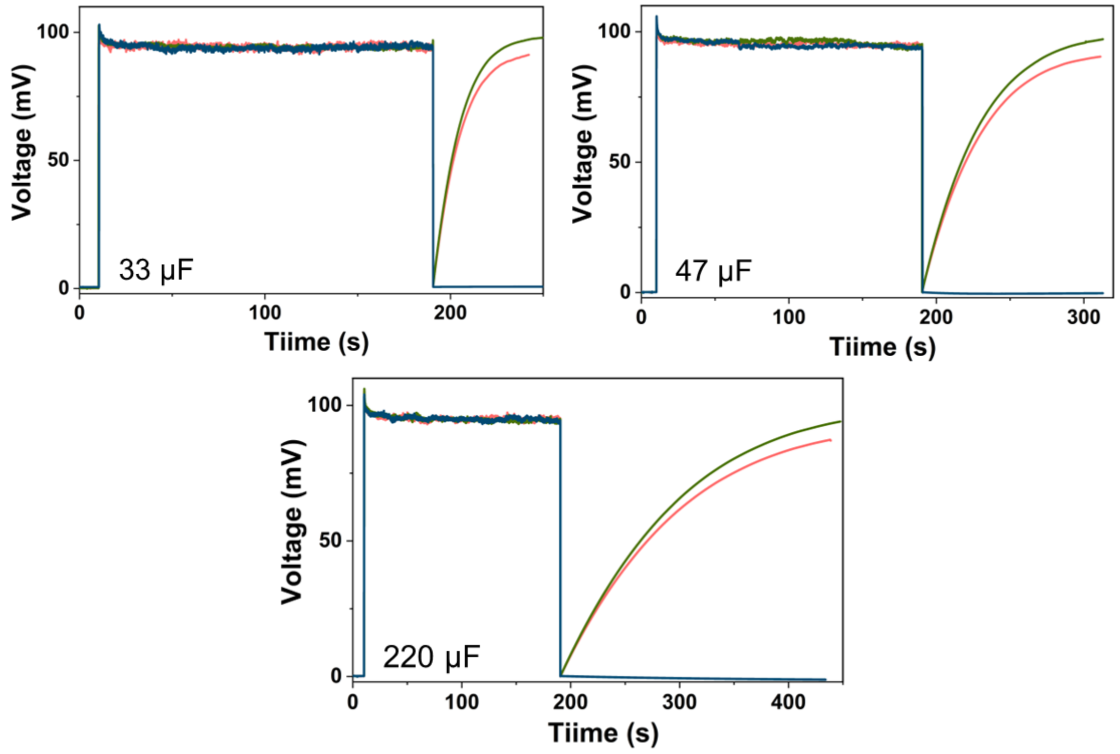

Fig. S22 The measured voltage curves of different capacitors under 450 nm light illumination (4.5 mW/cm^2)

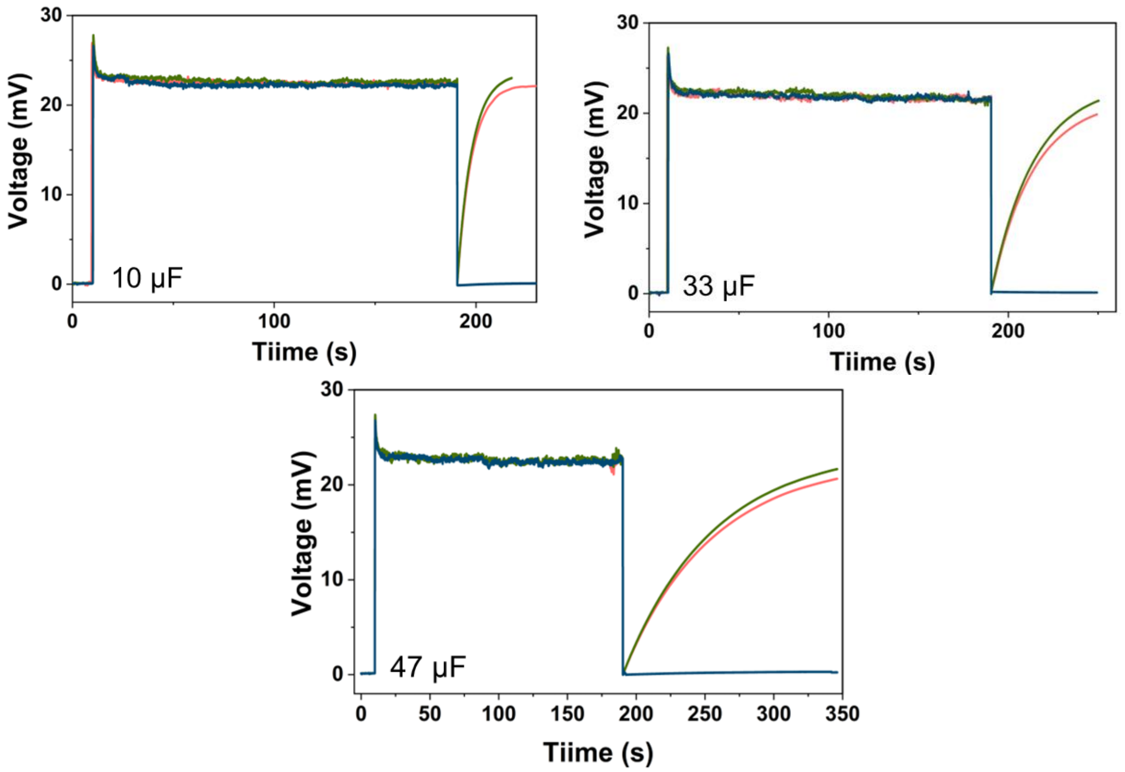

Fig. S23 The measured voltage curves of different capacitors under 515 nm light illumination (4.5 mW/cm^2)

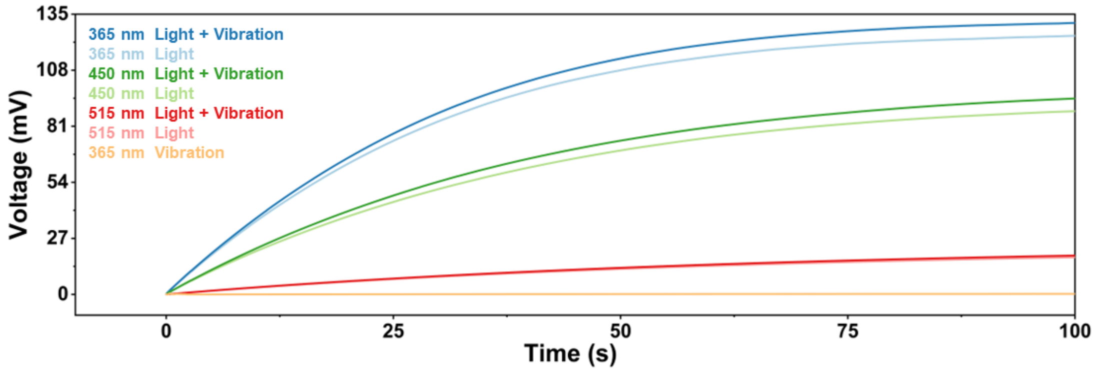

Fig. S24 The measured voltage curves of the nanogenerator for the same capacitor (47 μF) under different wavelengths light in the same time (4.5 mW/cm^2)

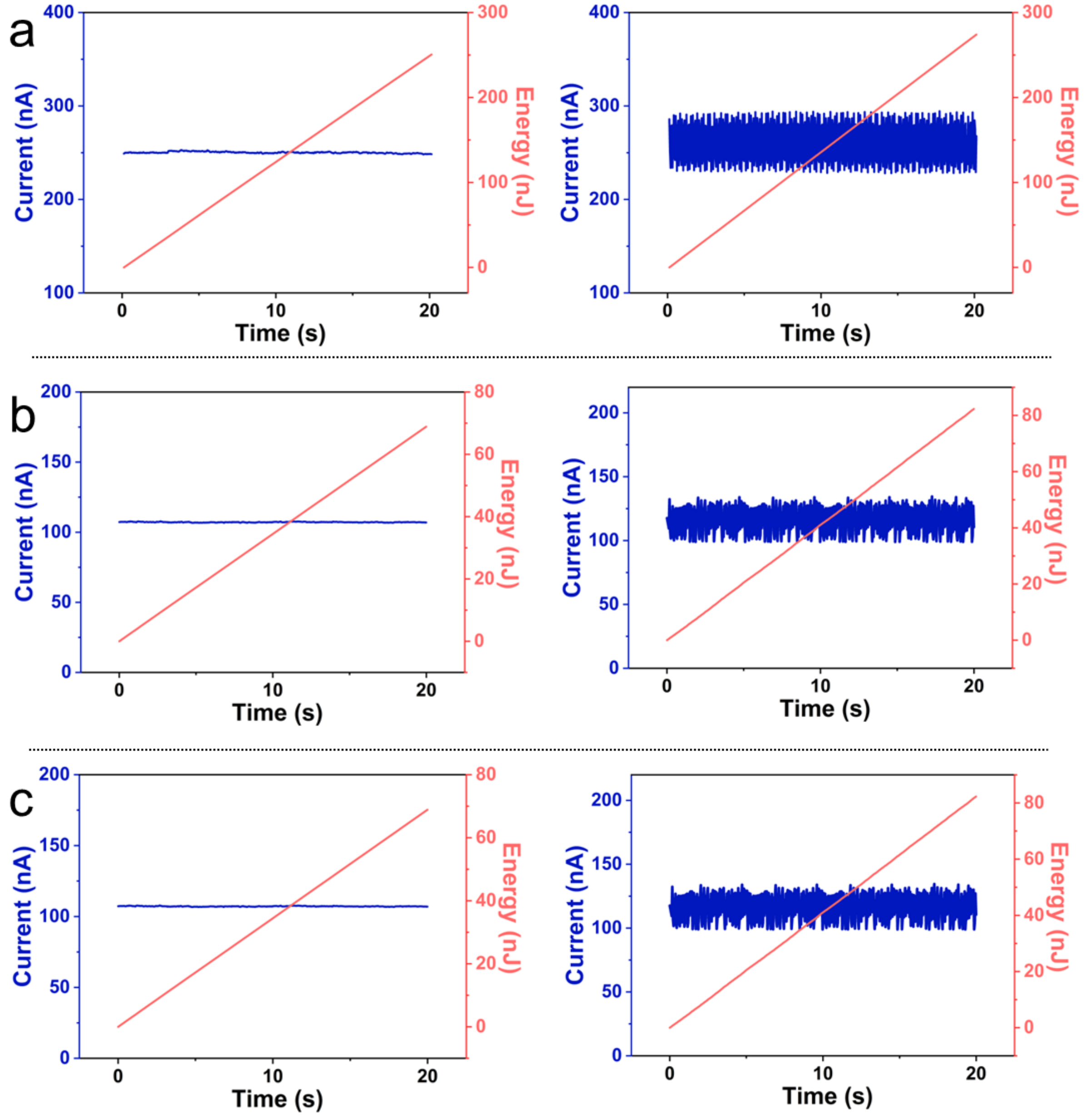

Fig. S25 Current-energy curves. Photo-current and coupling current under (**a**) 365 nm, (**b**) 450 nm and (**c**) 515 nm light illumination and energy integration curves for the corresponding time periods

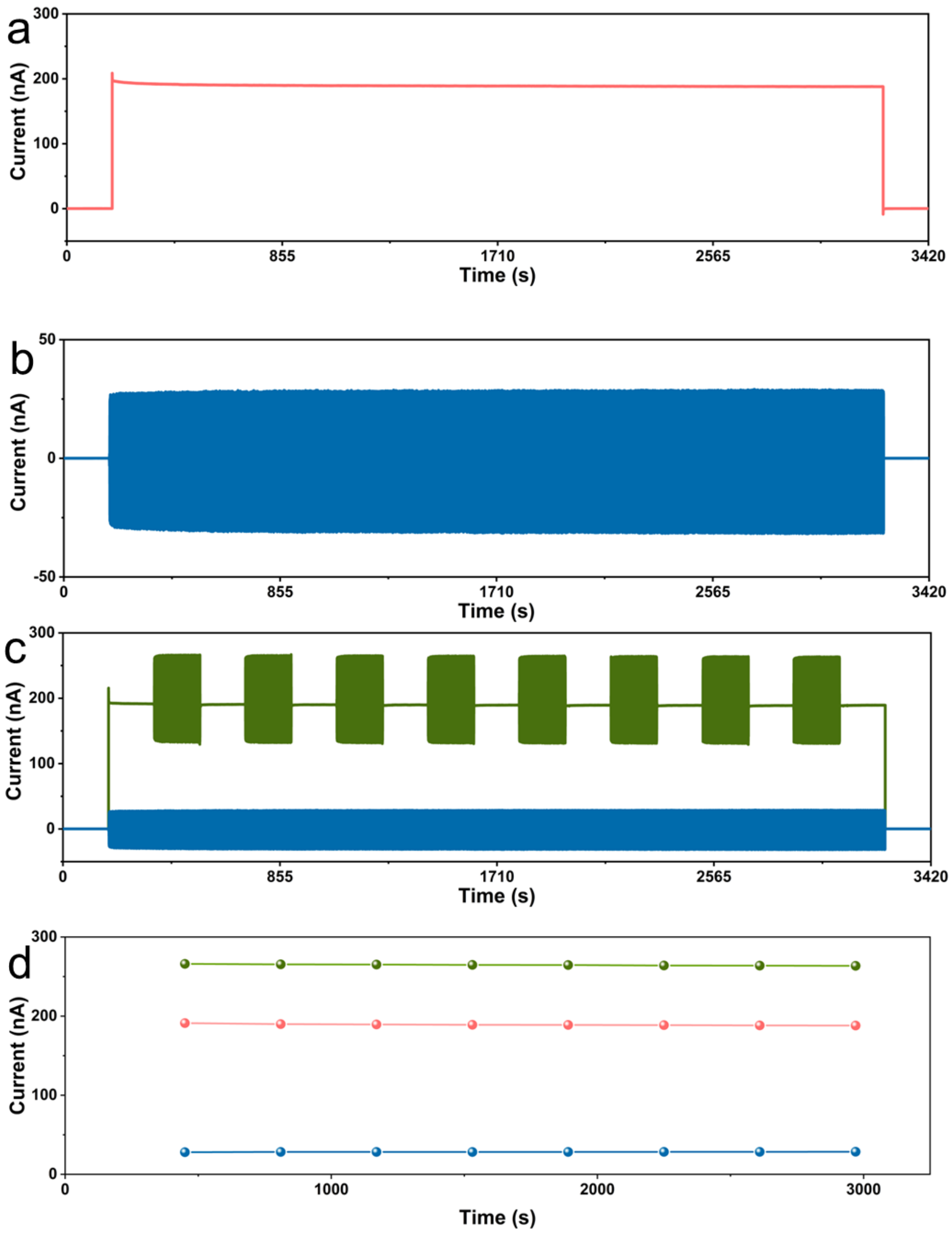

Fig. S26 Stability testing of devices. (**a**) Photocurrents. (**b**) Flexoelectric currents. (**c**) Coupling current. (**d**) Statistical results for a, b and c

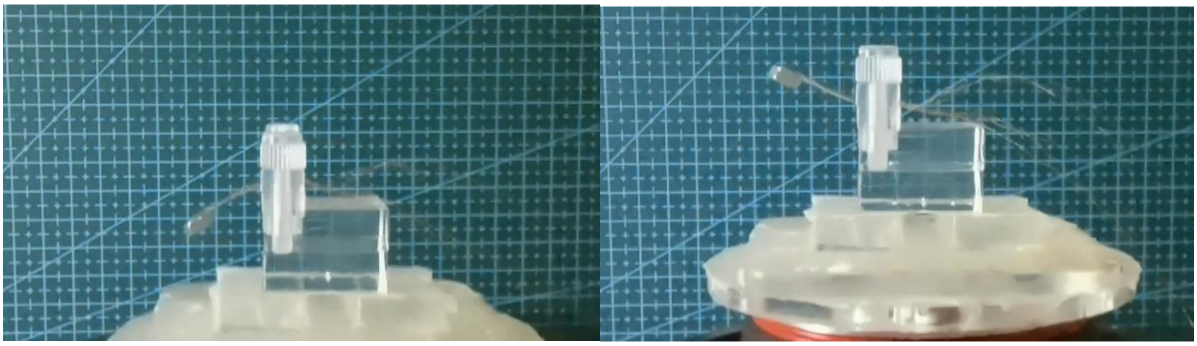

Fig. S27 The optical images of the bending of the device during operation
